# A globally distributed durophagous marine reptile clade supports the rapid recovery of pelagic ecosystems after the Permo-Triassic mass extinction

**DOI:** 10.1038/s42003-022-04162-6

**Published:** 2022-11-14

**Authors:** Yu Qiao, Jun Liu, Andrzej S. Wolniewicz, Masaya Iijima, Yuefeng Shen, Tanja Wintrich, Qiang Li, P. Martin Sander

**Affiliations:** 1 School of Resources and Environmental Engineering, Hefei University of Technology, 193 Tunxi Road, Hefei, 230009 China; 2 Section Paleontology, Institute of Geosciences, University of Bonn, Nussallee 8, 53115 Bonn, Germany; 3 Institute of Paleobiology, Polish Academy of Sciences, Twarda 51/55, 00-818 Warsaw, Poland; 4 Nagoya University Museum, Furocho, Chikusa-Ku, Nagoya, Aichi 464-8601 Japan; 5 Department of Comparative Biomedical Sciences, The Royal Veterinary College, North Mymms, Hertfordshire AL9 7TA United Kingdom; 6 The Dinosaur Institute, Natural History Museum of Los Angeles County, Los Angeles, CA 90007 USA

**Keywords:** Palaeontology, Phylogenetics

## Abstract

Marine ecosystem recovery after the Permo-Triassic mass extinction (PTME) has been extensively studied in the shallow sea, but little is known about the nature of this process in pelagic ecosystems. Omphalosauridae, an enigmatic clade of open-water durophagous marine reptiles, potentially played an important role in the recovery, but their fragmentary fossils and uncertain phylogenetic position have hindered our understanding of their role in the process. Here we report the large basal ichthyosauriform *Sclerocormus* from the Early Triassic of China that clearly demonstrates an omphalosaurid affinity, allowing for the synonymy of the recently erected Nasorostra with Omphalosauridae. The skull also reveals the anatomy of the unique feeding apparatus of omphalosaurids, likely an adaptation for feeding on hard-shelled pelagic invertebrates, especially ammonoids. Morphofunctional analysis of jaws shows that omphalosaurids occupy the morphospace of marine turtles. Our discovery adds another piece of evidence for an explosive radiation of marine reptiles into the ocean in the Early Triassic and the rapid recovery of pelagic ecosystems after the PTME.

## Abstract (web summary)

Further evidence for an explosive marine reptile diversification event in the Early Triassic is presented via morphological analysis of a new *Sclerocormus* specimen.

## Introduction

Marine ecosystem recovery after the Permo-Triassic mass extinction (PTME), the most severe extinction in the history of the Earth, has been traditionally regarded as delayed and gradual^1,2^. However, this view has been challenged by an increasing number of recent discoveries suggesting the rapid emergence and evolution of Mesozoic marine reptiles in the aftermath of the PTME^3–8^. Marine reptiles attained high taxonomic and ecomorphological diversity as early as the late Early Triassic, and included generalist predators^9^, piscivores^10^, specialized forms using non-visual prey detection^11^, lunge feeders^6^ and durophages^12^. Durophagy evolved independently in several clades of Triassic marine reptiles, including ichthyosauriforms (the recently erected nasorostrans and several groups of ichthyopterygians)^13,14^, placodonts^15^ and thalattosaurs^16^, but these durophagous ecomorphs were mostly limited to shallow-water environments and likely fed on abundant, sessile and benthic hard-shelled invertebrates. In fact, our understanding of the ecosystem recovery after the PTME is in general heavily biased towards data from shallow-water environments^17^ and relatively little is known about the nature of the recovery in open-water ecosystems^3^.

Ammonoids, a now extinct group of open marine cephalopods, had a successful ecological and evolutionary history for >300 million years during the Palaeozoic and Mesozoic, but their evolution experienced a severe bottleneck, with only three genera surviving the PTME^18^. Immediately after the mass extinction, however, ammonoids diversified explosively in the first few million years, and continued to play a significant ecological role for the rest of the Mesozoic due to their abundance, wide distribution, and high evolutionary rates^18^. Despite their high abundance in Mesozoic marine ecosystems, little is known about predation on ammonoids^19^. While sharks, mosasaurs, and cephalopods have been hypothesized as major groups that preyed upon ammonoids in the Jurassic and Cretaceous oceans, information regarding predators on Triassic ammonoids is scanty^19^.

Omphalosauridae, comprising several species in the genus *Omphalosaurus*, is an enigmatic group of durophagous marine reptiles known hitherto from Early–Middle Triassic pelagic sediments of Western North America^20,21^, Svalbard^22^, and the Bavarian Alps^23^. There is one exception where a single jaw fragment was found in the Middle Triassic shallow marine carbonates of Southern Poland, but this occurence is likely a stray carcass^24^. Omphalosaurids are characterised by the presence of durophagous dentition arranged in unique dental batteries both in the upper and lower jaws, in which functional teeth underwent extreme tooth wear leading to almost complete tooth loss before shedding^23^. Even though omphalosaurids were first reported over a century ago^20^, they are represented in the fossil record mostly by jaw fragments and only a few specimens preserving partial cranial and postcranial remains have been discovered to date^20,23^. As a consequence, the taxonomic affinity of omphalosaurids has remained elusive, with several groups of Mesozoic reptiles, including rhynchosaurs, placodonts and ichthyosaurs, proposed as their closest relatives^20,21,23^. The incomplete nature of omphalosaurid fossil material, as well as some stark anatomical differences between them and other groups of Mesozoic reptiles, have hindered their unambiguous placement in a phylogenetic context and their role in pelagic ecosystem recovery after the PTME.

Here, we describe a new specimen of the basal ichthyosauriform *Sclerocormus*^25^ from the Early Triassic of China. Its skull and dentition share numerous synapomorphies with those present in *Omphalosaurus* and another basal ichthyosauriform –  *Cartorhynchus*^13^. Our phylogenetic analysis unambiguously establishes omphalosaurids as early diverging ichthyosauriforms and necessitates the synonymy of the recently erected Nasorostra (comprising *Sclerocormus* and *Cartorhynchus*) with Omphalosauridae. The skull of the new specimen also reveals the anatomy of the bizarre feeding apparatus of omphalosaurids, suggesting the presence of a grinding mechanism unique among Triassic marine reptiles, that probably evolved as an adaptation for feeding on hard-shelled pelagic invertebrates, especially ammonoids. The peculiar feeding ecology of omphalosaurids likely enabled these reptiles to become geographically widespread rapidly following the PTME event, suggesting the early establishment of complex and functional trophic webs in pelagic waters.

## Results

### Systematic palaeontology

Diapsida Osborn, 1903

Ichthyosauromorpha Motani et al., 2015

Ichthyosauriformes Motani et al., 2015

Omphalosauridae Merriam, 1906 (=Nasorostra Jiang et al., 2016)

### Definition

The last common ancestor of *Omphalosaurus nevadanus, Cartorhynchus lenticarpus* and *Sclerocormus parviceps* and all of its descendants.

### Revised diagnosis

Omphalosauridae is differentiated from all other Ichthyosauriformes by the presence of the following synapomorphies: elongate nasal reaching the snout tip; maxilla excluded from the border of the external naris; crushing dentition forming irregular maxillary and dentary batteries concentrated along cranial midline; dome-shaped tooth crown; first maxillary and dentary teeth rounded and blunt; premaxilla edentulous; convex occlusal surface of maxilla and corresponding concave occlusal surface of dentary; deep posterior mandible with slanting end and low jaw joint; wing-like process on posterior dentary.

*Sclerocormus* Jiang et al., 2016

### Revised diagnosis

Skull small; tail long; trunk short and deep; preorbital snout constricted and short; preorbital and postorbital parts of skull subequal in length; postorbital extending backward to the posterior edge of the upper temporal fenestra; parietal posteriorly bifurcating into two prongs; coracoid larger than scapula; humerus and femur straight; radius and ulna subequal in length; gastralia robust and comprising three sets, each gradually tapering towards their lateral ends.

*Sclerocormus* cf. *parviceps* Jiang et al., 2016

### Referred specimen

**Fig. 1:**
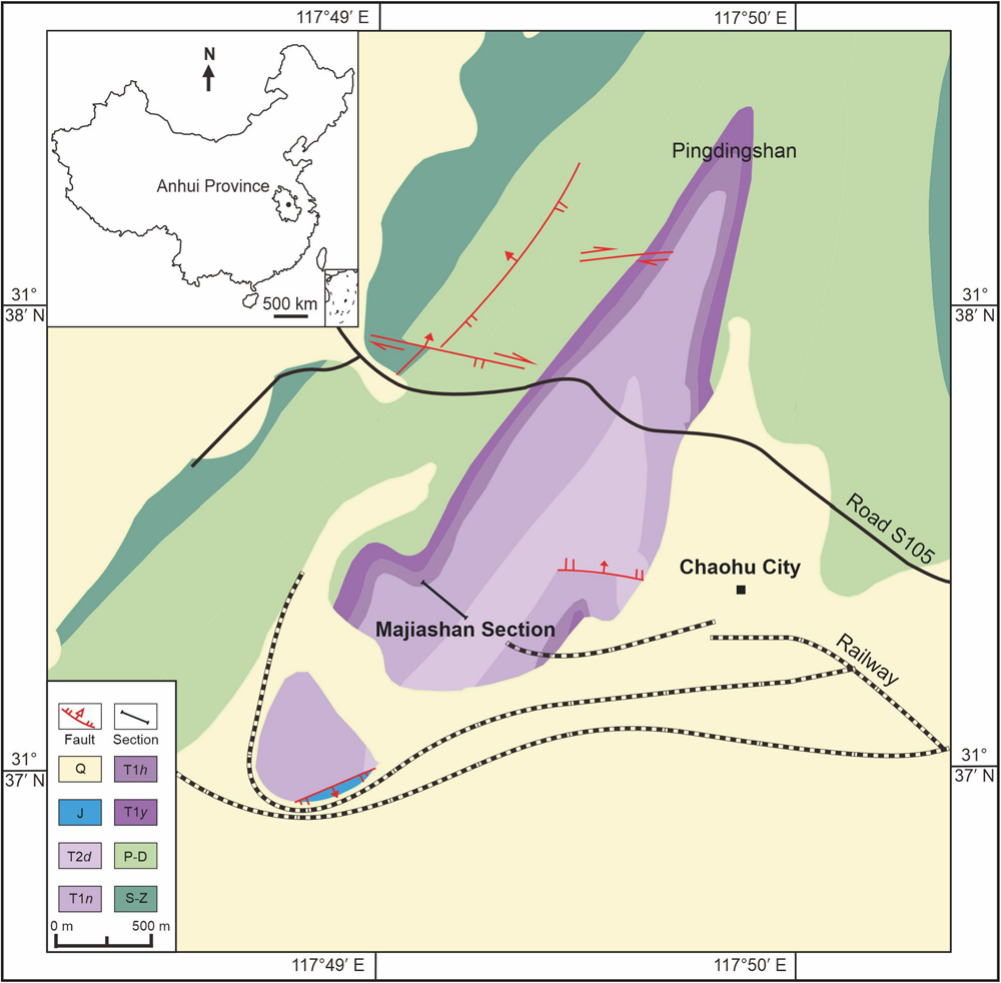
Geological map (updated after ref. 50) showing the locality of the Majiashan Section, Chaohu, Hefei, Anhui Province, China. Inset is the map of China. Abbreviations: Q Quaternary, J Jurassic, T2*d* Dongmaanshan Formation, T1*n* Nanlinghu Formation, T1*h* Helongshan Formation, T1*y* Yinkeng Formation, P-D Permian-Devonian, S-Z Silurian-Sinian.

**Fig. 2:**
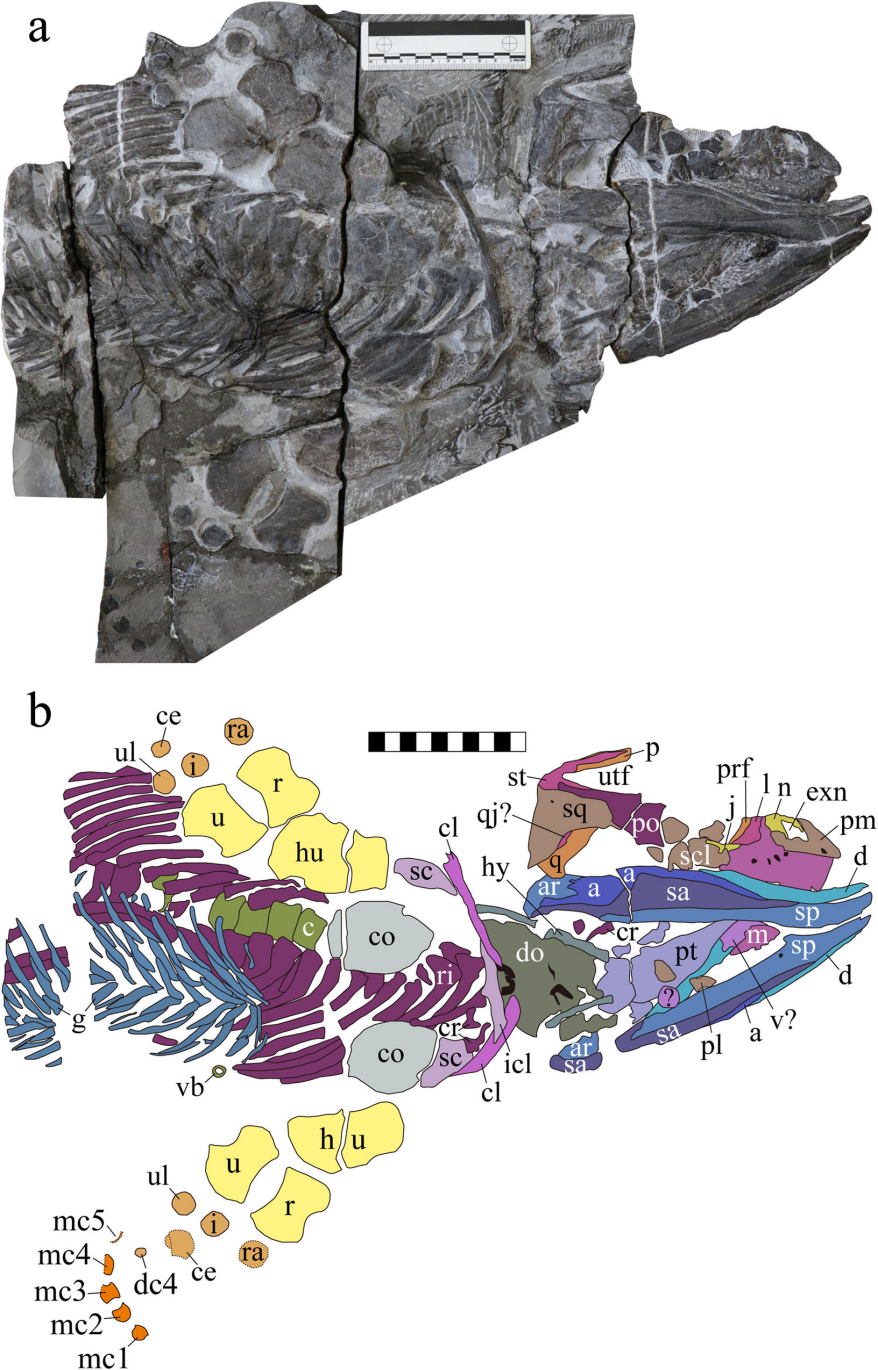
The skeleton of *Sclerocormus* cf. *S. parviceps* (HFUT MJS-16-012). **a** Photograph showing the ventral view of the skeleton. **b** Interpretative drawing. Abbreviations: a angular, ar articular, c centrum, ce centralia, cl clavicle, co coracoid, cr cervical rib, d dentary, dc distal carpal, do dermal ossicles, ec ectopterygoid, exn external naris, g gastralia, hu humerus, hy hyoid, i intermedium, icl interclavicle, j jugal, l lacrimal, m maxilla, mc metacarpal, n nasal, pl palatine, pm premaxilla, po postorbital, prf prefrontal, pt pterygoid, q quadrate, qj quadratojugal, r radius, ra radiale, ri ribs, sa surangular, sc scapula, scl scleral ossicles, sp splenial, sq squamosal, st supratemporal, u ulna, ul ulnare, utf upper temporal fenestra, v vomer. Scale bars equal 10 cm.

**Fig. 3:**
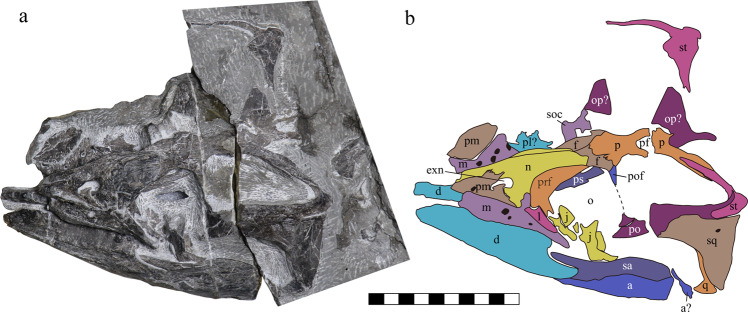
The skull of *Sclerocormus* cf. *S. parviceps* (HFUT MJS-16-012). **a** Photograph showing the left lateral view of the skull. **b** Interpretative drawing. Abbreviations: a angular, d dentary, exn external naris, f frontal, j jugal, l lacrimal, m maxilla, n nasal, o orbit, op opisthotic, p parietal, pf pineal foramen, pl palatine, pm premaxilla, po postorbital, pof postfrontal, prf prefrontal, ps parasphenoid, q quadrate, sa surangular, soc supraoccipital, sq squamosal, st supratemporal. Scale bar equals 10 cm.

HFUT MJS-16-012, a partial skeleton housed at Hefei University of Technology (HFUT). The new specimen was collected from Majiashan quarry in Chaohu, Hefei, Anhui Province, China (Fig. 1). The fossiliferous horizon is from the Upper Member of the Nanlinghu Formation, Spathian, Olenekian, Lower Triassic (~248 Ma), representing an open water environment (Supplementary Figs. 1, 2; Supplementary Results). The specimen was largely prepared from ventral view, but the skull was also prepared from both lateral sides as much as possible (Figs. 2, 3). Measurements of HFUT MJS-16-012 are available in Supplementary Results and Supplementary Table 1, respectively. The new information available from HFUT MJS-16-012 also allows us to re-interpret the morphology of *Omphalosaurus* cf. *O. nevadanus* (MBG 1500; see Supplementary Fig. 4 and Supplementary Results) - a key specimen of *Omphalosaurus*^23^ to compare with the Chinese specimens.

### Morphological remarks

HFUT MJS-16-012 is referred to *Sclerocormus* on the basis of its large body size compared to the co-occurring *Cartorhynchus*^13^, as well as a well-developed gastral basket and ossified centralia that clearly differentiate it from *Cartorhynchus*^13^. The skull length of HFUT MJS-16-012 cannot be accurately measured since the tip of the snout is damaged. However, the upper and lower jaws are well articulated with each other in HFUT MJS-16-012 (Figs. 2, 3), and there is no apparent overbite present in the *Cartorhynchus* holotype^13^, the only known omphalosaurid specimen with well-preserved and articulated upper and lower jaws. Therefore, we can safely estimate the skull length of HFUT MJS-16-012 by measuring it from the tip of the lower jaw to the posterior margin of the supratemporal. This gives a minimum estimate of the skull length of 226.7 mm for HFUT MJS-16-012 since the tip of the lower jaw is also slightly damaged. Thus, we estimate that the HFUT MJS-16-012 skull is more than twice larger than the skull of the holotype of *S. parviceps* (skull length = 100 mm)^25^. Using the simple skull-body length proportion of the holotype specimen, the total length of HFUT MJS-16-012 is estimated to have reached as much as 3.6 m. However, HFUT MJS-16-012 might still not be fully mature as the carpal region is poorly ossified. Whether the stark difference in body size between HFUT MJS-16-012 and the holotype of *S. parviceps* is due to taxonomic or intraspecific variation remains uncertain. HFUT MJS-16-012 also possesses a proportionally smaller orbit than the holotype specimen (1/4 of skull length, compared with 1/3 for the holotype), a frontal contribution to the orbit (frontal excluded from orbital margin in holotype), three rows of gastralia (two rows of gastral elements reported in holotype), and one centralium and one distal carpal preserved in the forefin (in contrast to two centralia and two distal carpals preserved in the holotype). The decrease in the relative size of the orbit throughout ontogeny has been demonstrated in ichthyopterygians and other reptiles^26^, so a similar phenomenon in *Sclerocormus* seems likely. Furthermore, the skull of the holotype specimen is dorsolaterally compressed. This is likely to have an effect on the perceived differences in the relative contributions of the frontals to the orbital margin between both specimens. Because the gastral basket in the holotype specimen is largely disarticulated and scattered, it cannot be excluded that when articulated, the gastralia would have also formed three rows of elements. Finally, the numbers of ossified forefin elements have been demonstrated to vary in the forefin of the basal ichthyosauriform *Chaohusaurus*^9^, so the differences in the number of centralia and distal carpals in both specimens of *Sclerocormus* likely represent intraspecific variation. Because HFUT MJS-16-012 differs notably from the holotype only in its larger body size, we refrain from erecting a new species and tentatively refer the new specimen to *S*. cf. *S. parviceps*, pending a detailed comparative study between HFUT MJS-16-012 and the holotype.

**Fig. 4:**
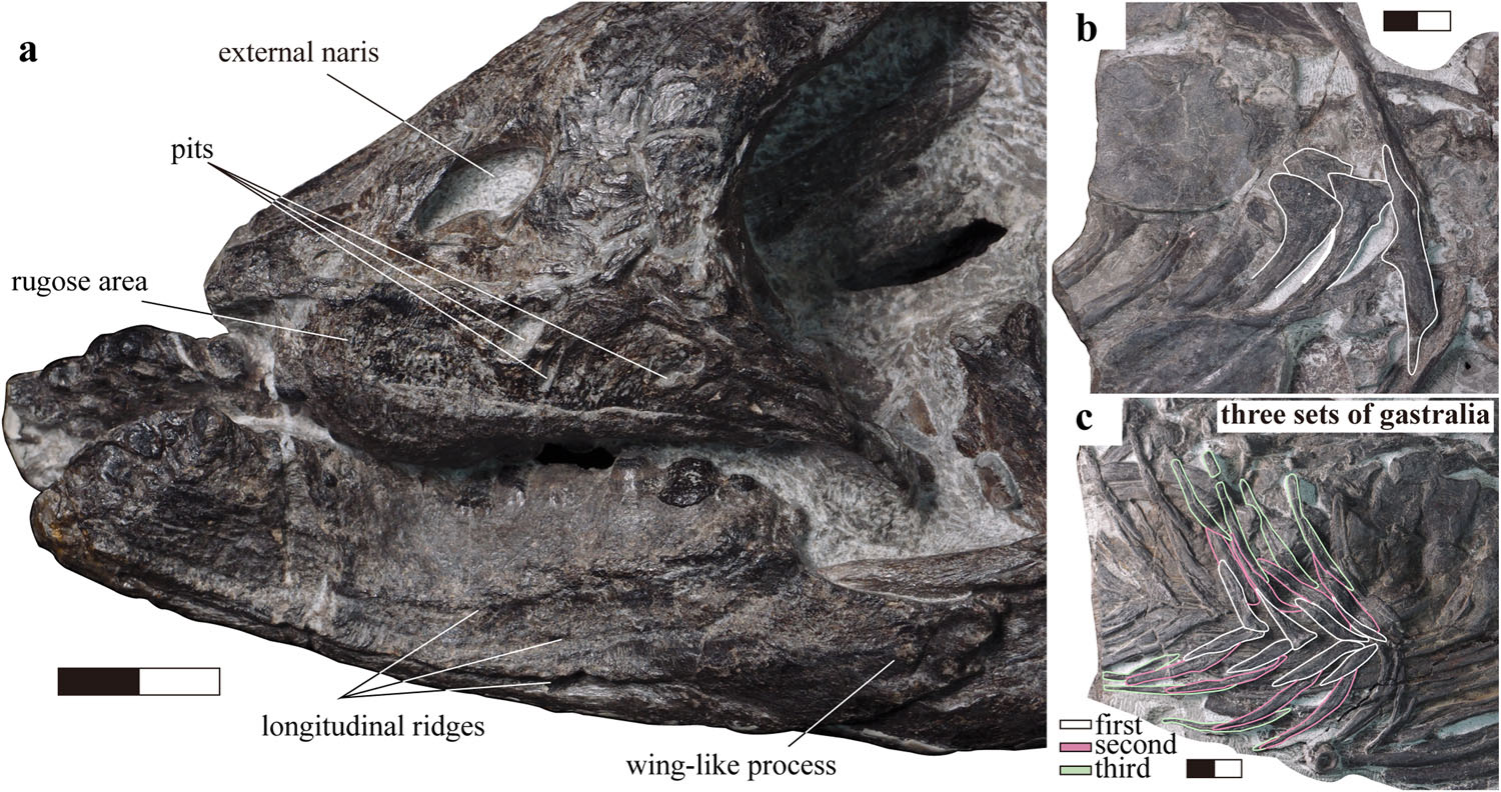
Details of the skeleton of *Sclerocormus* cf. *S. parviceps* (HFUT MJS-16-012). **a** Photo showing the surface ornamentation of the premaxilla, maxilla and dentary. **b** Photo showing the single-headed ribs and the interclavicle with a short posterior process. **c** Robust gastralia with three pairs of segments. Scale bars equal 2 cm.

**Fig. 5:**
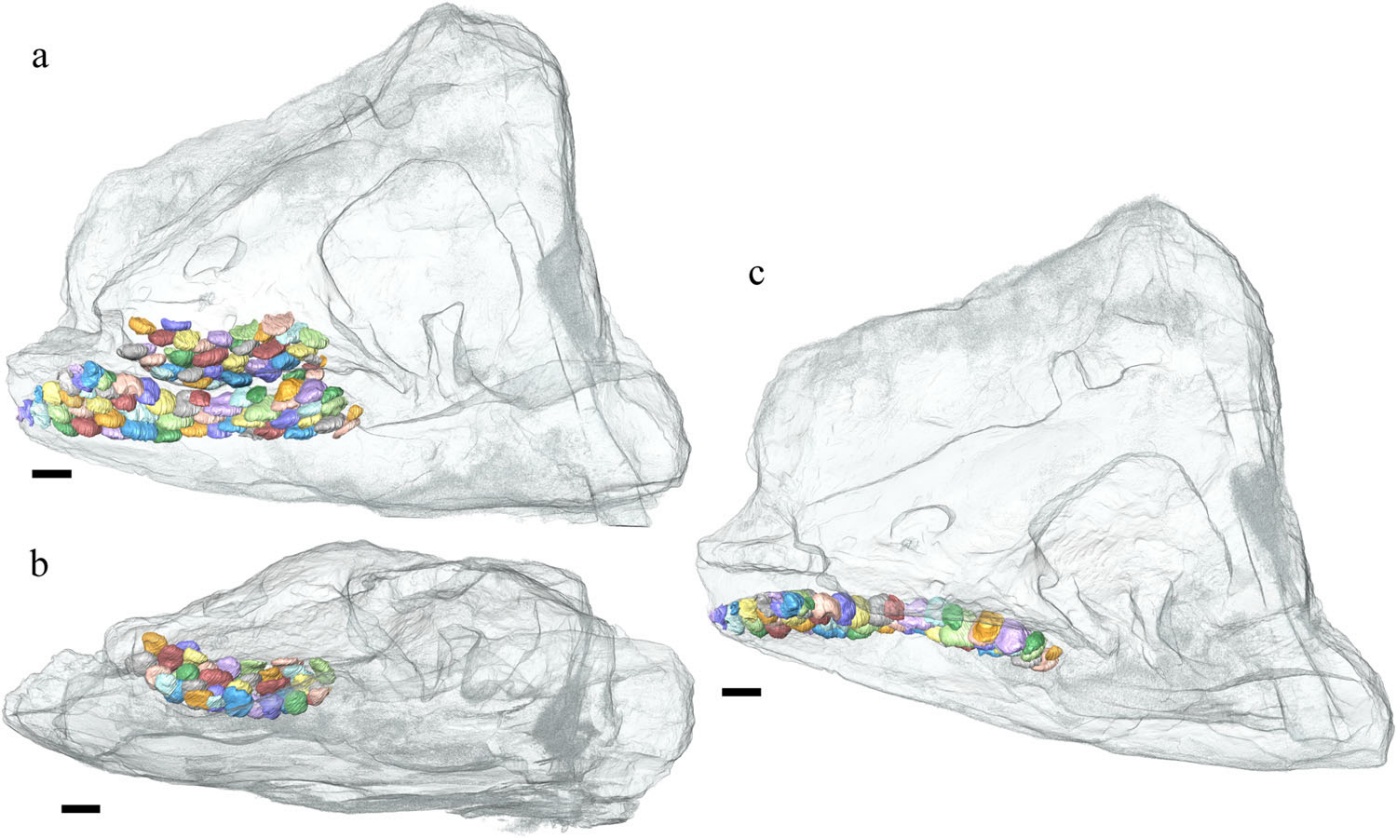
CT reconstruction showing the tooth arrangement in *Sclerocormus* cf. *S. parviceps* (HFUT MJS-16-012). **a** Maxillary dentition and dentary dentition in left lateral view. **b** Maxillary dentition in ventral view. **c** Dentary dentition in dorsal view. Scale bars equal 1 cm.

The comparison of HFUT MJS-16-012 with the holotype and only specimen of the sympatric *Cartorhynchus*^13^ is also of relevance. The exposed area of the quadrate is much smaller than the squamosal in HFUT MJS-16-012, although it is partially covered by the squamosal (Fig. 3). Conversely, the size of the quadrate and squamosal is not much different in *Cartorhynchus*^14^. The length of the upper temporal fenestra is significantly longer than that of the orbit in *Sclerocormus*, unlike the much smaller fenestra in *Cartorhynchus*^13^. The symphysis of HFUT MJS-16-012 is long, accounting for nearly 1/3 of the entire lower jaw length, while it is weak and much shorter (1/5 of the lower jaw length) in *Cartorhynchus*^14^. The splenials of HFUT MJS-16-012 meet within the symphysis and extend to the tip of the jaw, while they do not in *Cartorhynchus*^14^. *Cartorhynchus* has three functional tooth rows on the right dentary^14^, and no dental battery^23^, while HFUT MJS-16-012 only has a single functional tooth row on the lower jaw but has a dental battery in both the upper and lower jaws (Figs. 4a, 5). The interclavicle is anteriorly flat with a small process extending posteriorly in HFUT MJS-16-012 (Fig. 2), yet the bone is cruciform in *Cartorhynchus*. The number of presacral vertebrae in *Sclerocormus* is higher than that of *Cartorhynchus* (34 vs 31). The gastralia are arranged into three sets in HFUT MJS-16-012 and are robust, curved, and flat with grooves on the surface (Figs. 2, 4c), but they have a slender rod-like shape in *Cartorhynchus*^13^.

Distal carpal 4 of *Sclerocormus* is ossified (Fig. 2). However, there are no distal carpals preserved in the *Cartorhynchus* holotype^13^. The holotype of *Cartorhynchus* preserves an almost complete scleral ring, but the aperture of the scleral ring occupies <20% of the entire orbital area. Finally, the neural spines in *Cartorhynchus* are unfinished in dorsal view^13^. The above characteristics indicate that the *Cartorhynchus* holotype likely represents a juvenile individual^27^, rather than an adult as previously stated, but it is unlikely to be a juvenile ontogenetic stage of *Sclerocormus*.

**Fig. 6:**
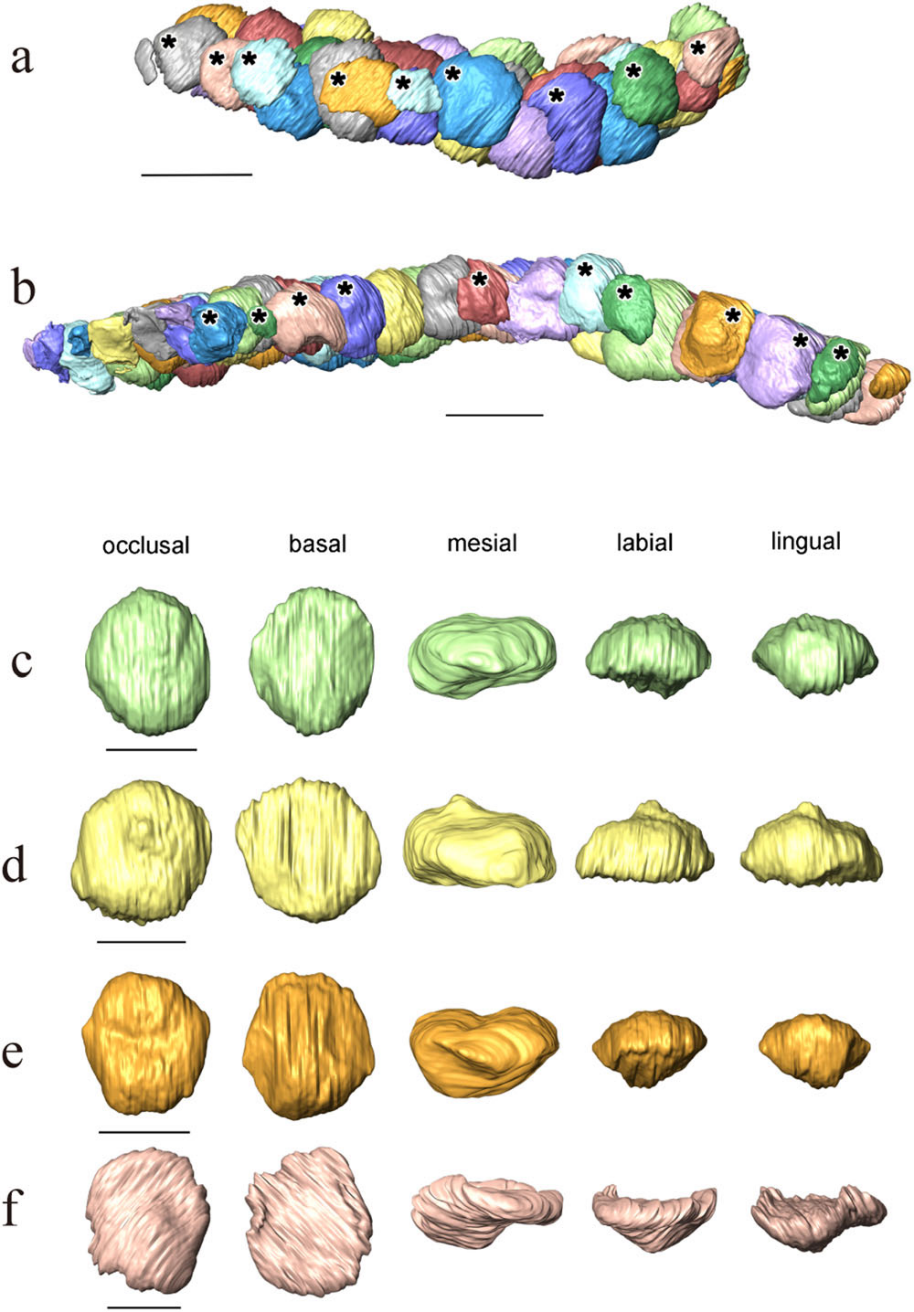
CT images showing the tooth shape of *Sclerocormus* cf. *S. parviceps* (HFUT MJS-16-012). **a**, **b** The maxillary and dentary tooth rows, respectively, in occlusal view; functional (surface) teeth are marked with asterisks; note that several functional teeth are heavily worn. **c**–**f** Some replacement teeth which exemplify the major morphological features displayed by the dentition. **c** Typical “dome shaped” occlusal surface of a dentary tooth. **d** The pointed apex on a dentary tooth. **e** The longitudinal groove on a dentary tooth. **f** An apico-basally thin replacement cone located posterodorsally in the maxilla, consisting almost entirely of enamel; note the irregular margin and reduced root. The scale bars for the tooth rows are 1 cm and the scale bars for each tooth are 5 mm.

**Fig. 7:**
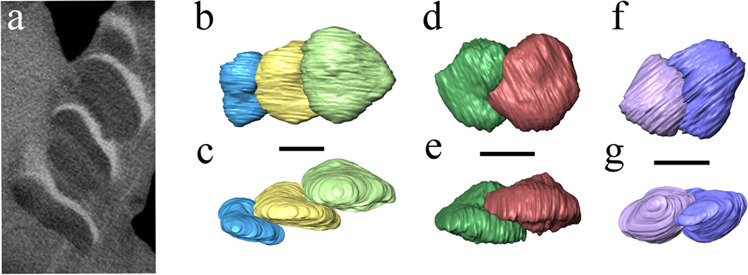
CT images showing the tooth histology and resorption of *Sclerocormus* cf. *S. parviceps* (HFUT MJS-16-012). **a** One slice showing the dental histology; white is the enamel cap, dark is dentin, and faint and light-grey bands are formed by incremental lines in dentin. **b**–**g** CT images showing the ways in which the teeth contact each other. **b**, **c** Posteroventral dentary teeth. **d**, **e** Anteroventral dentary teeth. **f**–**g** Posterior maxillary teeth. The scale bars are 5 mm.

**Fig. 8:**
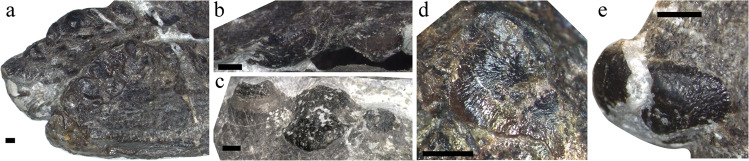
Photo showing the tooth details of *Sclerocormus* cf. *S. parviceps* (HFUT MJS-16-012). **a** Dentary dentition in left lateral view. **b** Maxillary dentition in ventral view. **c** Dentary dentition in dorsal view. **d** Close-up of the enamel surface morphology. **e** The tip of the first maxillary tooth. Note the ‘orange-peel-like’ pitting of the enamel surface in **b**, **c** and **e**. Scale bars equal 2 mm.

Although no complete skull is known for *Omphalosaurus*, the skull and the snout were likely short as suggested by *O. nettarhynchus*^28^, which is consistent with *Cartorhynchus* and *Sclerocormus* (Figs. 2, 3). The dentary in *Cartorhynchus* and *Sclerocormus* ends posteriorly in a characteristic wing-shaped extension that is raised slightly above the jaw surface (Fig. 4a) and is similar to the *Omphalosaurus nevadanus* holotype^20^ and isolated dentaries of *Omphalosaurus* sp. from Spitsbergen^22^. The deep and robust splenials, which are similar to those in *Omphalosaurus nevadanus*^20,23,28^, reach the tip of the jaw in HFUT MJS-16-012 (Fig. 2). The tooth-bearing parts in the Chinese specimens are distinctly longer in the lower jaw than the upper jaw (Fig. 5)^14^, as in the Alpine specimen of *Omphalosaurus*^23^. In HFUT MJS-16-012, the tooth rows are irregularly arranged (Fig. 5) and hard to define as in the Spitsbergen and Alpine *Omphalosaurus*^22,29^. A similar tooth morphology is shared among the new specimen of *Sclerocormus* (Figs. 4a, 5–8), *Cartorhynchus*, and *Omphalosaurus*: low dome-shaped crowns and multiple irregular tooth rows, while only *Sclerocormus* and *Omphalosaurus* share obvious ‘orange-peel-like’ pits on the enamel surface (Fig. 8)^14,23,24,29,30^.

### Phylogenetic analysis

**Fig. 9:**
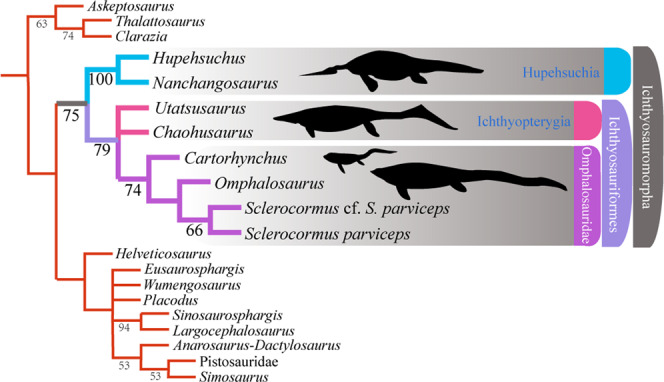
Simplified version of the strict consensus of eight most parsimonious trees obtained from the matrix of 47 taxa and 220 characters. Bootstrap support values (>50%) are shown below the nodes.

To test the phylogenetic position of *Sclerocormus*, *Cartorhynchus* and *Omphalosaurus* (scoring based mostly on *O. nevadanus*^20^ and *O*. cf. *O*. *nevadanus*^23^), we included these taxa into a modified phylogenetic matrix focusing on the relationships among diapsid reptiles (Supplementary Data 1, 2). Heuristic searches of the diapsid data matrix found eight most parsimonious trees (tree length = 897, consistency index = 0.297, retention index = 0.62). The strict consensus tree with bootstrap nodal support values is shown in Fig. 9 and Supplementary Fig. 3. Our phylogenetic analysis recovered all three taxa as forming a sister clade to Ichthyopterygia, nested within Ichthyosauriformes. Several dental and cranial characters shared between *Omphalosaurus*, *Cartorhynchus* and *Sclerocormus* confirm their monophyly and support the synonymy of the recently erected Nasorostra with Omphalosauridae (see Systematic Palaeontology).

### Functional morphospace analysis

**Fig. 10:**
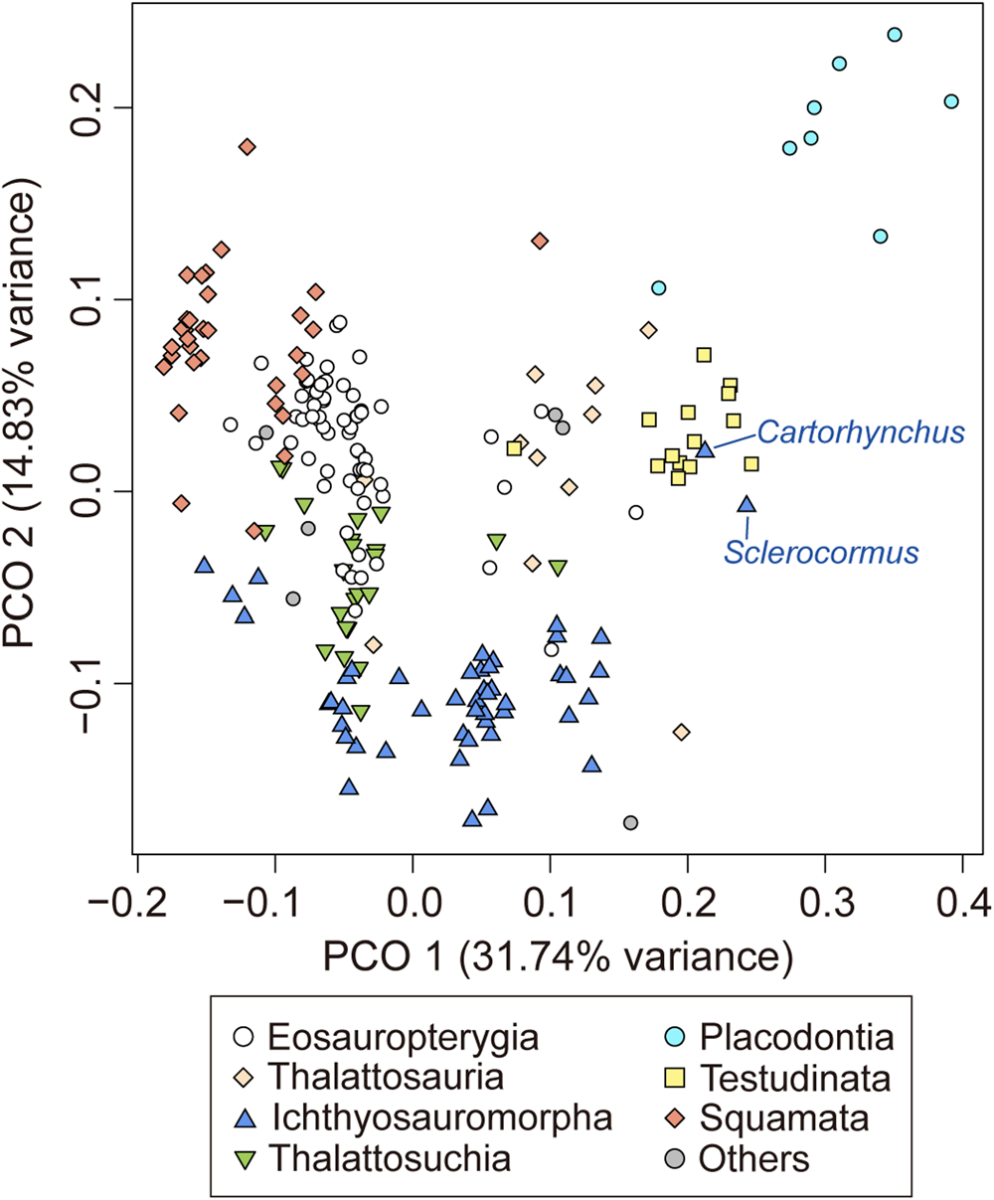
Functional morphospace showing the distribution of Mesozoic marine reptiles.

To understand the ecology of omphalosaurids, we performed a functional morphospace analysis based on a revised data matrix (Supplementary Data 3, 4)^7^. PCO 1 and 2 explain nearly 50% of the craniodental morphological variation among Mesozoic marine reptiles. Morphospaces of durophagous forms such as placodonts, the durophagous mosasaur *Globidens*, and the omphalosaurid *Cartorhynchus* and *Sclerocormus* are convergently shifted towards higher PCO 1 and 2 values within each subclade (Sauropterygia, Squamata and Ichthyosauromorpha). However, *Cartorhynchus* and *Sclerocormus* do not occupy the morphospace of shallow-marine durophagous marine reptiles, but instead plot beyond the edge of the ichthyosauromorph space among that of marine turtles (Fig. 10).

## Discussion

*Cartorhynchus* and *Sclerocormus*, originally assigned to Nasorostra, were previously not considered as omphalosaurids because their dentition was not exposed in the holotypes^13,25^. Even though durophagous dentition was later identified in *Cartorhynchus*^14^, detailed comparisons with the dentition of *Omphalosaurus* were not made, and the similarity between other characters of the skull to those of *Omphalosaurus* also went unnoticed. The new specimen of *Sclerocormus* demonstrates that Nasorostra are synonymous with Omphalosauridae, as evidenced by the tooth morphology, arrangement and replacement (Figs. 4a, 5–8), as well as the presence of an edentulous premaxilla and a wing-like process of the posterior dentary (Figs. 2–4), which are shared between *Sclerocormus*, *Cartorhynchus* and *Omphalosaurus*. Addition of the new specimen into a phylogenetic analysis also provides unambiguous evidence for the ichthyosauriform affinity of omphalosaurids, which are the sister clade to Ichthyopterygia (Fig. 9). The new specimen also supports the taxonomic distinctness of *Cartorhynchus* and *Sclerocormus*, because the number of presacral vertebrae is different between them^14,25^, and the interclavicle in the new specimen possesses only a short process extending posteriorly (Figs. 2, 4b), yet the bone is cruciform in shape in *Cartorhynchus*^13^. Such a marked difference in morphology is unlikely to be the result of ontogenetic changes. Currently, Omphalosauridae is represented by three genera spanning the Early–Middle Triassic^13,23,25^. However, the co-occurrence of two genera in Majiashan quarry alone, and the morphological differences present between different species of *Omphalosaurus* indicate that the generic diversity of Omphalosauridae is likely underestimated. In fact, two different omphalosaurid morphotypes are also present in the Early Triassic of Svalbard – smaller specimens with teeth bearing clearly differentiated roots, resembling the dentition of *Cartorhynchus*, and larger specimens with rootless teeth, resembling the dentition of *Sclerocormus*^22^. It is uncertain, however, whether these two morphotypes represent intraspecific variability or taxonomic diversity.

The three-dimensionally preserved skull and mandible of the new specimen of *Sclerocormus* also allow for the reconstruction of the bizarre, specialised feeding apparatus of omphalosaurids for the first time. Prey capture was probably facilitated by the pointed tips of the upper and lower jaws, and the prey was transported towards the oral cavity perhaps with the aid of suction^31^, although the hyoids are weakly developed in *Sclerocormus* (Fig. 2). The seemingly durophagous functional dentition of omphalosaurids was not concentrated posteriorly, unlike in most other Triassic marine reptiles^12^. The lower jaw itself was slender and lacked a well-developed coronoid process, in contrast to the robust lower jaws of placodonts^32^ and the durophagous mosasaur *Globidens*^33^. Furthermore, the rugose surfaces of the maxillae and dentaries bear sharp ridges and pits (Fig. 4a), similar to those in the early turtle *Eorhynchochelys*^34^ that probably indicate the presence of a keratinous beak, that would have aided in the processing of hard-shelled prey. Our morphofunctional analysis demonstrates that the lower jaws of omphalosaurids were most similar to those of marine turtles (Fig. 10), which also possess a keratinous beak but lack teeth, and suggests omphalosaurid jaws were capable of capturing pelagic prey and processing a wide range of food items like pelagic foraging turtles^35–37^. The presence of heavily worn and frequently replaced teeth also suggests that omphalosaurids at least partially fed on abrasive hard-shelled invertebrates, especially ammonoids that were found in the same pelagic sediments as omphalosaurids^23^ (Supplementary Fig. 2).

Our phylogenetic analysis, which demonstrates that *Cartorhynchus* was the sister-clade of (*Sclerocormus* + *Omphalosaurus*), seems to indicate that throughout their evolution, the teeth of omphalosaurids underwent root reduction. An efficient grinding surface formed by numerous teeth dispersed along the occlusal surfaces of the jaw bones likely allowed omphalosaurids to process their food even more efficiently^23^. Given the lack of modern analogues of the feeding apparatus of omphalosaurids and modern open-water ecosystems dominated by hard-shelled invertebrates, an analysis of feeding and lifestyle in omphalosaurids inherently involves much speculation.

The dentition and feeding apparatus of omphalosaurids differ strikingly from those of other contemporaneous marine reptiles with blunt teeth that were restricted largely to more shallow-marine environments and likely fed on sessile and benthic invertebrates^12^. Therefore, we argue that omphalosaurids represented a unique lineage of pelagic predators capable of feeding on hard-shelled invertebrates. Even though the body plan of omphalosaurids lacked anatomical features associated with efficient pelagic cruising, they were likely well adapted to a pelagic lifestyle as evidenced by their highly cancellous bone histology, since the disappearance of compact bone in tetrapods is generally a manifestation of more aquatic adaptation^23^. Furthermore, omphalosaurids do not need to be efficient pursuit predators, as they could have fed on high-density but low-speed pelagic invertebrates such as ammonoids^38,39^. The fact that omphalosaurids are known almost exclusively from pelagic deposits in which they co-occur with ammonoids also supports our hypothesis.

*Cartorhynchus* was previously determined to be at least semi-terrestrial on the basis of its highly unossified flippers, low dorsal vertebral count, and relatively weak visual capacity^13^. However, strongly unossified flippers are also present in the likely fully aquatic basal ichthyosauriform *Chaohusaurus*^9^ and the vertebral count of *Cartorhynchus* also overlaps with the range for some other marine reptiles^13^. In addition, the seemingly weakly developed underwater vision of *Cartorhynchus* could still have been useful for detecting pelagic invertebrates, especially in the photic zone^13^. Therefore, *Cartorhynchus* is probably a pelagic animal like other omphalosaurids, as also supported by the deep water environment of Majiashan quarry (Supplementary Results; Supplementary Figs. 1, 2).

Omphalosauridae was the most widespread clade of ichthyosauriforms of their times, now known from all important Northern Hemisphere pelagic regions that have produced Early and Middle Triassic marine reptiles, in contrast to other groups of ichthyosauriforms with more restricted distribution in time and space^40^. Their evolution seems to have tracked the fast recovery of ammonoids^41^ and conodonts^42^ in the Early and Middle Triassic. The prevailing view on marine ecosystem recovery after the PTME has been that the shallow-water Middle Triassic biotas rich in sauropterygians are representative for the fully recovered ecosystems around the world^1^ and that the Early Triassic ichthyosauromorph domination was replaced by Middle Triassic sauropterygian domination^25^. Our study suggests that the radiation was not only amazingly rapid in shallow-water ecosystems^6^, but also in the open ocean, as indicated by the evolution of omphalosaurids and colossal macropredatory ichthyosaurs^3,43^. The broad geographic distribution and high taxonomic and ecomorphological diversity of Early and Middle Triassic ichthyosauriforms underscores their crucial role in the ecosystem recovery after the PTME, adding further evidence to the emerging scenario of rapid ecosystem recovery in its aftermath in the open ocean^18^.

## Methods

### μCt scan of the skull

The new specimen (HFUT MJS-16-012) was scanned with a micro X-ray computed tomography scanner (NIKON XTH 320/225 LC: 200 kV and 180 mA, voxel size = 0.146 × 0.146 × 0.146 mm) at the X-Ray Computed Tomography and Multi-Scale Simulation Laboratory, School of Civil Engineering and Architecture, Zhejiang University, Hangzhou, China. A total of 3142 slices were generated. The original slices were read into ImageJ 1.52a^44^ and edited using the Brightness/Contrast tool in order to enhance the contrast between the dental tissues and the surrounding bone and to reduce the effect of beam-hardening. The edited slices were then uploaded into Avizo 2019.4 (Thermo Fischer Scientific). The individual teeth were manually segmented using the Lasso tool.

### Phylogenetic analysis

We modified the recent taxon-character matrix for diapsids with a particular focus on marine reptiles^25^. The matirx was prepared using Mesquite. Two new characters were added to the original data matrix. Character scores for eight taxa (Parareptilia, *Helveticosaurus*, *Placodus*, *Largocephalosaurus*, *Sinosaurosphargis*, *Wumengosaurus*, *Simosaurus* and Pistosauridae) were modified and Pachypleurosauria was replaced with an *Anarosaurus*-*Dactylosaurus* operational taxonomic unit (see Supplementary Data 1 for details). The new specimen (HFUT MJS-16-012) was included in the matrix independently of the holotype of *Sclerocormus*, and two more taxa, *Eusaurosphargis* and *Omphalosaurus*, were included as well. The coding of the holotypes of *Cartorhynchus* and *Sclerocormus* was updated based on the recent restudy^14^, and that of *Eusaurosphargis* was based on ref. ^45^. The coding of *Omphalosaurus* was based on the literature and direct specimen observations. See Supplementary Table 2 for referred specimens, and Supplementary Data 1 for the description of the new characters and the new coding. A heuristic analysis was performed in TNT 1.5 (random seed = 1, Wagner tree replicates = 5000, numbers of trees held per replicate = 10, branch swap algorithm = tree bisection and reconnection)^46^. All multistate characters were treated as unordered. A bootstrap sampling of 1000 replicates of the dataset was conducted to measure the nodal support. The NEXUS file is available in Supplementary Data 2.

### Functional morphospace analysis

A previous data matrix of 18 morphofunctional characters and 207 taxa (Supplementary Data 3)^7^ was modified for the morphospace analysis. Characters 4–6 were removed due to the subjectivity of assessing the extent of temporal musculature in extinct taxa. Measurements and coding for *C. lenticarpus* and *Sclerocormus* (HFUT MJS-16-012) were modified or newly added to the original dataset from published photographs and personal examination. Following the method described in ref. 7, raw continuous data were z-transformed, and a Gower’s distance matrix^47^ was generated from the combined continuous + discrete data, using the R package StatMatch^48^. The distance matrix was subjected to principal coordinate analysis (Supplementary Data 4) using the cmdscale function in R^49^.

### Reporting summary

Further information on research design is available in the Nature Research Reporting Summary linked to this article.

## Supplementary information

Supplementary Information

Description of Additional Supplementary Files

Supplementary Data 1

Supplementary Data 2

Supplementary Data 3

Supplementary Data 4

Reporting Summary

## Data Availability

All data needed to evaluate the conclusions in the paper are present in the paper and/or the Supplementary information files.

## Code availability

The authors declare that the code supporting the findings of this study is available in the Supplementary information files.
