# Supplementary material for: A globally distributed durophagous marine reptile clade supports the rapid recovery of pelagic ecosystems after the Permo-Triassic mass extinction
Source: Commun Biol. 2022 Nov 14;5:1242. doi: 10.1038/s42003-022-04162-6 (PMC9663502; doi:10.1038/s42003-022-04162-6)
Supplement: Supplementary file 1 — Supplementary Information [file 42003_2022_4162_MOESM1_ESM.pdf]

Supplementary Information for

A globally distributed durophagous marine reptile clade supports the rapid recovery of pelagic ecosystems after the Permo-Triassic mass extinction

Yu Qiao, Jun Liu*, Andrzej S. Wolniewicz, Masaya Iijima, Yuefeng Shen, Tanja Wintrich, Qiang Li, P. Martin Sander

*Corresponding author. Email: junliu@hfut.edu.cn

Table of Contents

Supplementary Results ········ 2
Geological Background ········ 2
Morphological reinterpretation of *Omphalosaurus* cf. *O. nevadanus* (MBG 1500) ········ 3
Supplementary Figs. ········ 5
Supplementary Tables ········ 10
Supplementary References ········ 12

Supplementary Results

Geological background. The specimen was collected from the second level of Majiashan quarry (31°37′13.67″N, 117°49′9.15″E), ~2 km NW from Chaohu, Hefei, Anhui Province, China (Fig. 1). The Majiashan Section is located on the western limb of the Pingdingshan syncline (Fig. 1). From base to top, the Majiashan Section exposes three Lower Triassic formations: the upper part of the Helongshan Formation, the Nanlinghu Formation, and the lower part of the Dongmaanshan Formation. This succession has a total thickness of about 230 meters.

The upper part of the Helongshan Formation is composed of greenish to grey thin-bedded to nodular calcareous mudrock and shale with some nodules and concretions of fishes and bivalves at the top (Supplementary Figs. 1, 2). This unit contains the *Neospathodus waageni* conodont zone, the *Anasibirites* ammonite zone, and the *Guichielle angulata* bivalve zone, indicating a late Smithian age (lower Olenekian)[1-10]. The Nanlinghu Formation is divided into three members. The Lower Member is composed of dark grey thick-bedded lime mudstone with grey thin-bedded marlstone and nodular limestone (Supplementary Figs. 1, 2). This member contains the *Neospathodus pingdingshanensis* and the *Neospathodus homeri* conodont zones, the *Columbites* to *Tirolites* ammonite zones, and the *Guichielle angulata* bivalve zone, indicating an early Spathian age (upper Olenekian)[1-10]. The Middle Member is composed of greenish to reddish nodular limestone and grey thin-bedded lime mudstone (Supplementary Figs. 1, 2). The Upper Member is composed of an intercalation of grey medium to thick-bedded lime mudstone and black calcareous shale, showing a shallowing upward sequence (Supplementary Figs. 1, 2). These two members contain the *Neospathodus anhuinensis* conodont zone, the *Procolumbites* and *Subcolumbites* ammonite zones, and the *Periclaraia circularis* bivalve zone, indicating a middle to late Spathian age (upper Olenekian)[1-11]. The lower part of the Dongmaanshan Formation is composed of light-grey to mauve medium to thick-bedded dolostone with birds' eyes, gypsum pseudomorphs, and dolomitic breccias at the top. This unit contains a series of fossils including conodont *Neospathodus longidentata*, bivalves *Asoella illyrica, Unionites* sp., *Chlamys* sp., as well as some gastropods, indicating an earliest Anisian age[12-13].

The Middle and Upper members of the Nanlinghu Formation have generated more than a hundred marine reptile specimens, catalogued collectively at Anhui Geological Museum, Peking University, and Hefei University of Technology. Except for one single specimen of *Chaohusaurus*[14], all other marine reptile specimens are from the Upper Member. The new specimen HFUT MJS-16-012 was also collected from the Upper Member of the Nanlinghu Formation by the late R.G. Cao, a passionate local fossil collector, and donated to HFUT in 2016. The exact fossil locality had already been covered by soil and trees when the specimen was donated, preventing the confirmation of the specific bed number. But the bed number of the new specimen can be confidently located between Beds 611-679 (Supplementary Fig. 1).

Petrographically, the fossiliferous horizons are composed of greenish to reddish nodular limestone and grey thin-bedded lime mudstone and an intercalation of grey

medium to thick-bedded lime mudstone, brownish marlstone and black calcareous shale (ribbon lime mudstone). There are also some slumps and climbing ripples occurring within the brownish marlstone (Supplementary Fig. 2). Based on the field observation and a combination of macro- and microfacies analysis[15-18] (Supplementary Fig. 2), a tranquil and relatively stagnant outer ramp to deep-water-basin environment could be inferred for the middle to upper part of the Nanlinghu Formation.

Morphological reinterpretation of *Omphalosaurus* cf. *O. nevadanus* (MBG 1500). Based on the new information obtained from HFUT MJS-16-012, we re-identified the unnamed bones A, B, C, and F of *Omphalosaurus* cf. *O. nevadanus* (MBG 1500; Supplementary Fig. 4) in ref.[19].

Bone A is boomerang-shaped with an oblique foramen near the tip of the ramus. One ramus of the convex margin is smooth and the other is serrated, indicating that it is connected to other bones. For the bones that make up the orbit, the convex margin including two rami of each bone is connected with other skull elements, so it is not the orbital bone. Besides, the lacrimal has a smaller curvature, while bone A has a much larger curvature, so it is not a lacrimal. Among the bones that surround the temporal fenestra and may have the shape of a boomerang are supratemporal and squamosal. In HFUT MJS-16-012, the squamosal also has a similar oblique foramen as in *Omphalosaurus* cf. *O. nevadanus*, which is located at the tip of the convex margin. So bone A is interpreted to be the squamosal here.

Bone B is boot-shaped. The angle formed by the concave margin of the two rami of this bone is more rounded, likely part of a skull opening. In basal ichthyosauromorphs, the bones with this characteristic are the prefrontal, postfrontal and jugal. The jugal is always slender in basal ichthyosauromorphs, but bone B appears robust. In the *Sclerocormus* holotype, the prefrontal and postfrontal are distorted, but in other basal ichthyosauromorphs including *Cartorhynchus*, *Chaohusaurus* and hupehsuchians, the postfrontal looks more like a boot compared with prefrontal[19-28]. Therefore, bone B may represent the postfrontal.

Bone C is elongated, with one side smooth and having a slightly convex margin, and the other side having a very convex process. In Chinese omphalosaurids, the nasal is elongated. The dorsal margin of nasal is slightly convex, and the ventral side has an obvious protrusion in the middle and articulates with the premaxilla, maxilla and prefrontal. The bone yields a ventral process, where the anterior and posterior sides of which forms the edge of the external naris and contacts the prefrontal, respectively. Among the other bones in the skull, there is no such bone with the characteristics of smooth convexity and anteroposteriorly elongated except for the nasal. So bone C must be the nasal.

Bone F is incomplete and partially compressed. It is slender and elongated with a longitudinal groove on the surface. The only bone with a slender rod-like shape and deep grooves or bifurcations in Chinese omphalosaurids is the parietal. So bone F may be the posterior part of the bifurcated parietal.

The tooth-bearing bones of the *Omphalosaurus*[19,29] were identified as

premaxillae. But the premaxilla is much smaller than the maxilla and yields a large number of teeth distributed on the convex occlusal surface in *Omphalosaurus*, similar to the maxilla of the new specimen. In Chinese omphalosaurids, it is very clear that there are no teeth in the premaxilla. So the bones identified as premaxillae in *Omphalosaurus* must be maxillae.

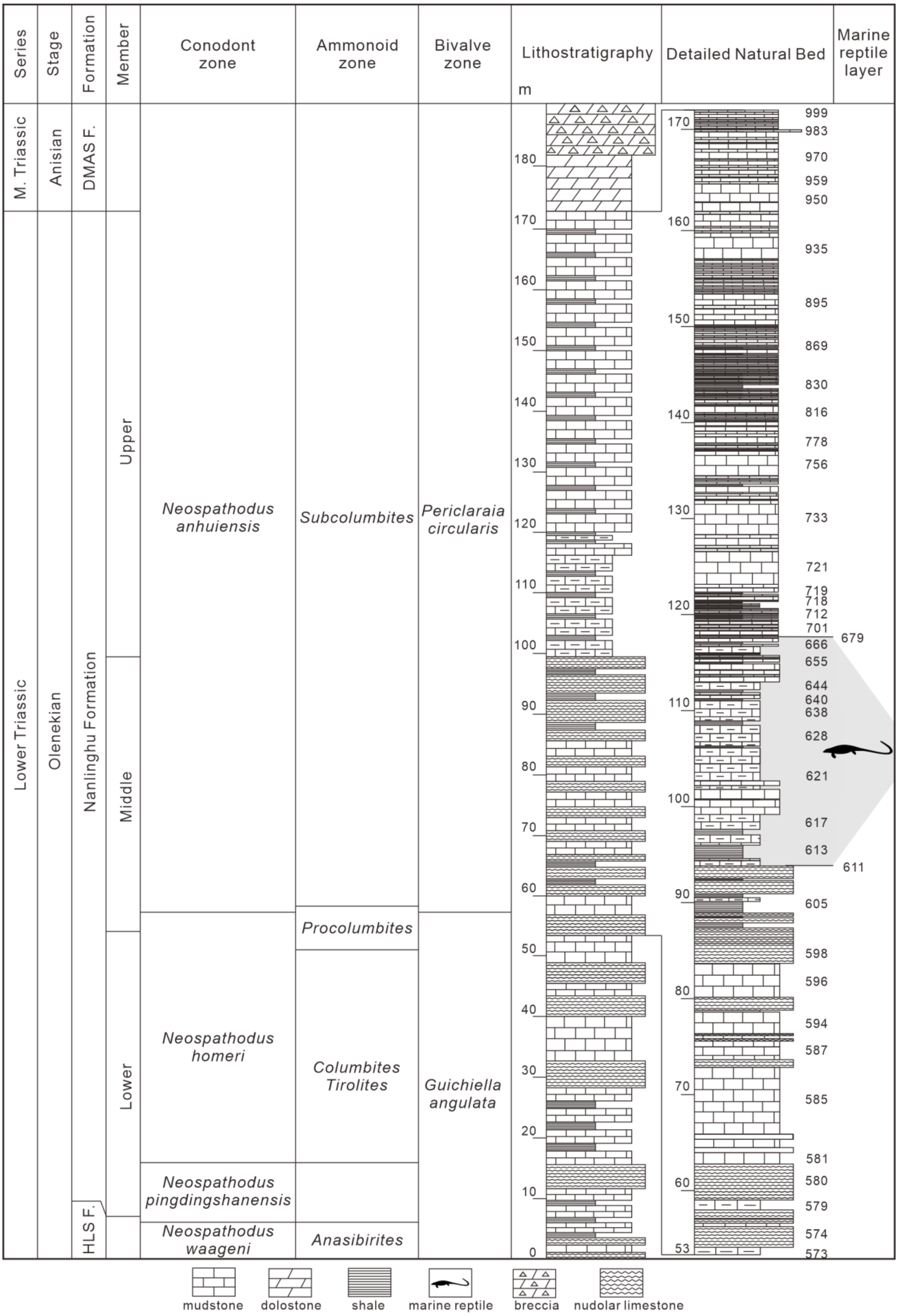

Supplementary Fig. 1 Comprehensive litho- and biostratigraphy of the Majiashan Section, Chaohu, Hefei, Anhui Province, China (modified after ref.[30]). Abbreviations: M. Triassic, Middle Triassic; DMAS F., Dongmaanshan Formation; HLS F., Helongshan Formation.

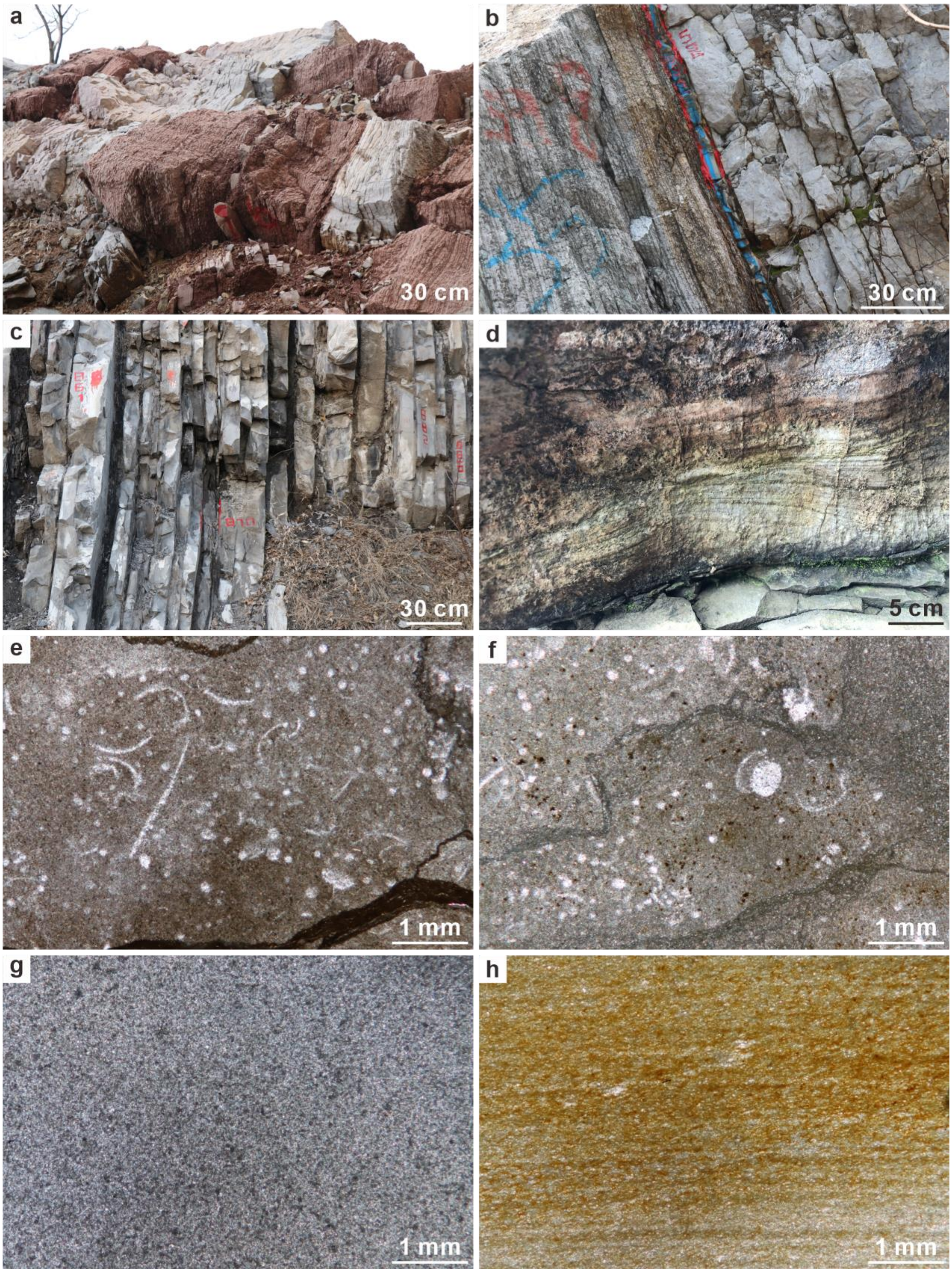

Supplementary Fig. 2 Field and thin-section petrography indicating a carbonate outer ramp to basin environment for the marine reptile-burying Middle and Upper members of Nanlinghu Formation. a-d Field photos. **e-h** Thin-section microphotographs. **a** Field photo showing intercalation of reddish nodular limestone and light grey thin-bedded lime mudstone, corresponding to the natural beds 574 to 578 in Supplementary Fig. 1. **b** Field photo showing the succession of greenish nodular limestone, and the intercalation of light grey thin-medium bedded lime mudstone and black calcareous shale, corresponding to the natural beds 596 to 598 in

Supplementary Fig. 1. **c** Field photo showing the intercalation of grey medium-thick bedded lime mudstone and black calcareous shale, corresponding to the natural beds 856 to 887 in Supplementary Fig. 1. **d** Field photo showing the climbing ripples at the boundary of black calcareous shale (lower part) and medium bedded lime mudstone (upper part) in Bed 612. **e** Thin-section microphotograph showing the bioclastic wacke-packstone microfacies for the reddish nodular limestone in Bed 574; note the abundant bioclastic fragments of pelagic calcispheres, thin-shelled ostracods and mollusks. **f** Thin-section microphotograph showing the bioclastic wacke-packstone microfacies for the greenish nodular limestone in Bed 595; note the abundant bioclastic fragments of pelagic calcispheres, thin-shelled ostracods, mollusks, sponge spicules and the axial section of an ammonoid (right center). **g** Thin-section microphotograph showing the calcisilt to mudstone microfacies of the grey medium-bedded ribbon limestone in Bed 593. **h** Thin-section microphotograph showing the laminated mudstone microfacies of the climbing ripple containing marlstone in Bed 594.

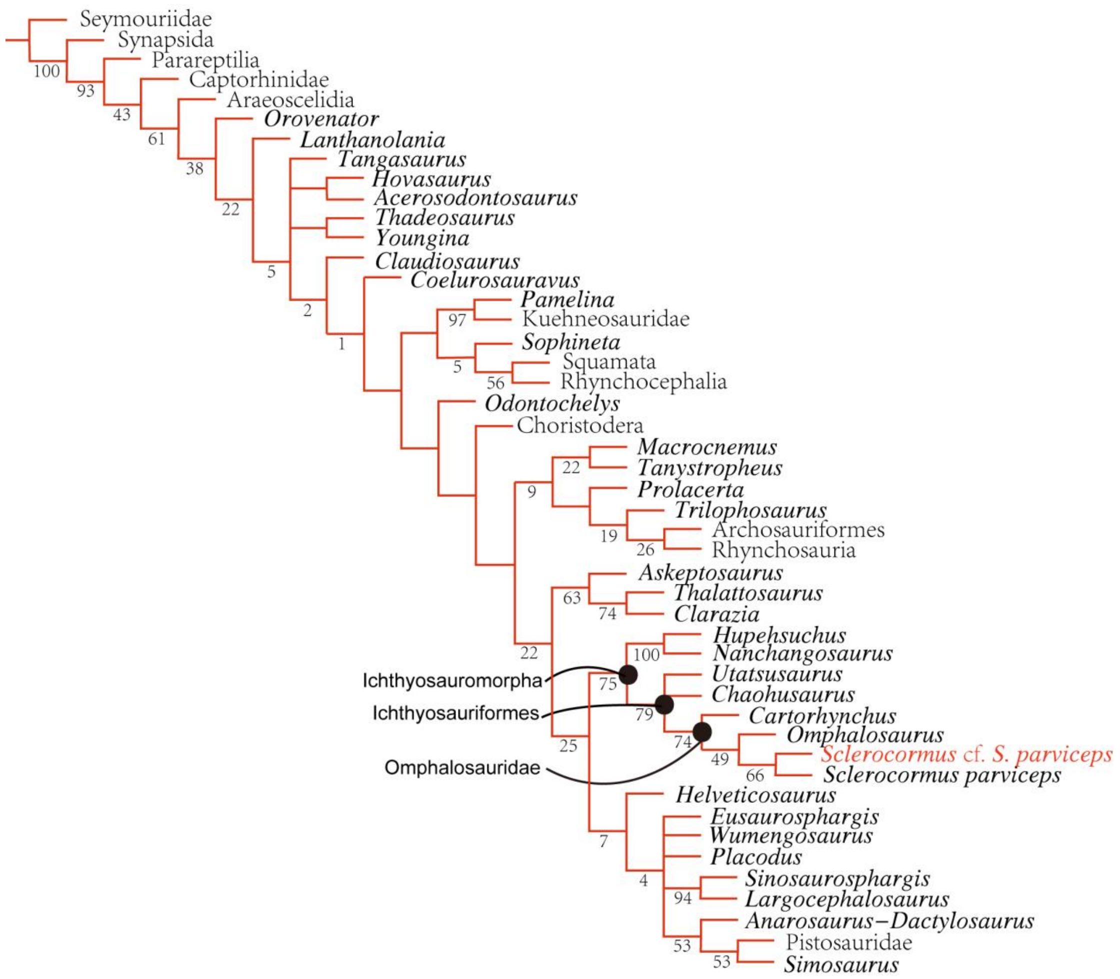

Supplementary Fig. 3 Phylogenetic hypotheses of *Sclerocormus* cf. *S. parviceps* (HFUT MJS-16-012) among Diapsida. A heuristic analysis was performed in TNT 1.5 (random seed = 1, Wagner tree replicates = 5000, numbers of trees held per replicate = 10, branch swap algorithm = tree bisection and reconnection). All multistate characters were treated as unordered. A bootstrap sampling of 1000 replicates of the dataset was conducted to measure the nodal support.

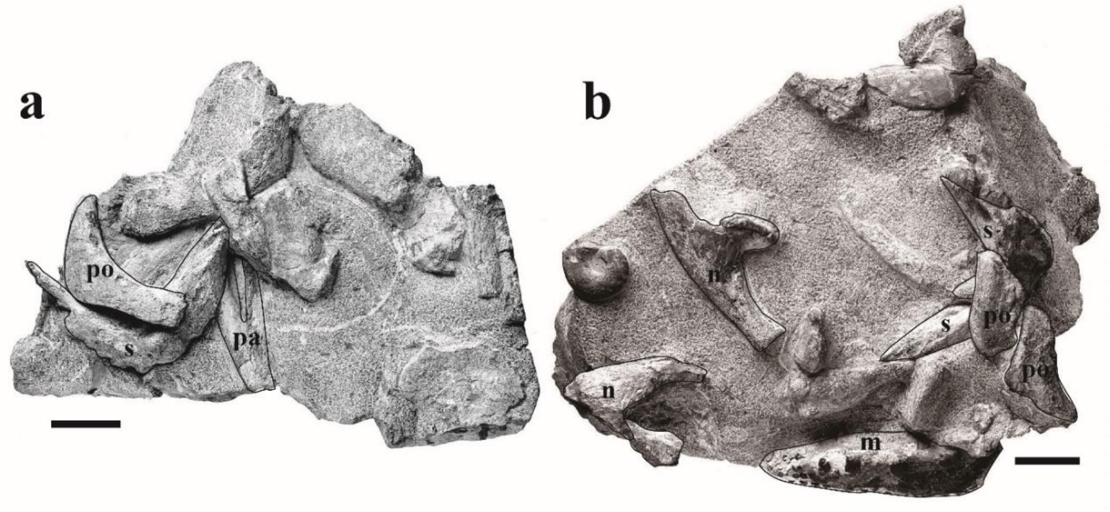

Supplementary Fig. 4 Reinterpretation of the skull of *Omphalosaurus* cf. *O. nevadanus* (MBG 1500)[19]. a Photograph of slab I of MBG 1500 and reinterpretation. **b** Photograph of slab II of MBG 1500 and reinterpretation. Abbreviations: m, maxilla; n, nasal; pa, parietal; po, postfrontal; s, squamosal. Scale bars are 5 cm.

Supplementary Table 1. Measurements (in mm) of the *Sclerocormus* cf. *S. parviceps* (HFUT MJS-16-012).

Length of skull along the mid-line	125.2+
Length of the prenarial snout	27.3+
Length of the premaxilla	45.6+
Length of the preorbital snout	65.1+
Length of the external naris opening	17
Length of the right lower jaw	218.8+
Length of the left lower jaw	229.1+
Anterior-posterior length of the orbit	45.5
Anterior-posterior length of the upper temporal fenestra	49.8+
Width of the parietal skull table at the level of pineal fossa	19.4+
Anterior-posterior length of the preserved largest dorsal vertebrate	17.5
Dorsal-ventral length of the preserved largest dorsal vertebrate	25.1
Length of the preserved largest rib head	24.0+
Length of the dorsal process of the right clavicle	92.9
Width of the lateral corner of the right clavicle	9.9
Length of the interclavicle	65.0
Length of the dorsal-ventral diameter of the scapula	34.5
Antero-posterior length of the scapula	44.6
Antero-posterior length of the coracoid	57.5
Width of the coracoid	45.6
Length of the preserved carpal space	103.6
Width of the waist of the left ulna	24.2
Width of the waist of the left radius	24.9
Width of the preserved distal end of the ulna	42.3
Largest diameter of the ulnare	15.5
Largest diameter of the preserved intermedium	19.1
Largest diameter of the radiale	19.2
Largest diameter of the centralia	13.5
Largest diameter of the 3rd? distal carpal	6.5
Length of 1st metacarpal	9.8
Length of 2nd metacarpal	11.1
Length of 3rd metacarpal	11.4
The largest crown width on the dentary	7.61
The smallest crown width on the dentary	4.18
The length of dentary	117.13

Supplementary Table 2. List of specimens compared in this paper and literature referred to. Abbreviations: AGB, Anhui Geological Museum, Hefei, China; GMPKU, Geological Museum of Peking University, Beijing, China; HFUT, Hefei University of Technology, Hefei, China; IVPP, Institute of Vertebrate Paleontology and Paleoanthropology, Beijing, China; MBG, Museum Burg Golling, Golling, Austria; MGL, Geological Museum of Lausanne, Lausanne, Switzerland; MHI, Muschelkalkmuseum Hagdorn, Ingelfingen, Germany; NA, not available; UCMP, University of California Museum of Paleontology, Berkeley, USA; UHR, Hokkaido University, Sapporo, Japan; WHGMR, Wuhan Institute of Geology and Mineral Resources.

**Taxa**	**Specimens**	**References**
Sclerocormus parviceps	AGB6265	25
Cartorhynchus lenticarpus	AGB6257	23, 31
Omphalosaurus nevadanus	UCMP 8281	32-33
Omphalosaurus sp.	MHI 1314; MHI 1829	34-35
Omphalosaurus nettarhynchus	MGL 45452	36
Omphalosaurus wolfi	MBG 1500	19, 34, 37
Omphalosaurus nisseri	Uncatalogued	29, 38
Hupehsuchus nanchangensis	IVPP V3232	39
Chaohusaurus brevifemoralis	AGB7401	28
Chaohusaurus chaoxianensis	GMPKU-P-3086; GMPKU-P-3188; IVPP V11362	26<br>40
Chaohusaurus zhangjiawanensis	WHGMR V26001; WHGMR V26025	20
Chaohusaurus geishanensis	IVPP V4001	40
Grippia longirostris	PMU R445	41
Utatsusaurus hataii	UHR 30691	42

Supplementary References

1. Tong, J.-N., Yuri, D. Z., Michael, J. O., Yin, H.-F. & Hans, J. H. A candidate of the Induan-Olenekian boundary stratotype in the Tethyan region. *Science in China Series D: Earth Sciences* **46**, 1182–1200 (2003).
2. Tong, J.-N., Zakharov, Y. D. & Wu, S. Early Triassic ammonoid succession in Chaohu, Anhui Province. *Acta Palaeontologica Sinica* **43**, 192–204 (2004).
3. Tong, J.-N., Hans, J. H., Zhao, L.-S. & Zuo, J.-X. High-resolution Induan-Olenekian boundary sequence in Chaohu, Anhui Province. *Science in China Series D: Earth Sciences* **48**, 291–297 (2005).
4. Tong, J.-N. & Zhao, L.-S. Lower Triassic and Induan-Olenekian Boundary in Chaohu, Anhui Province, South China. *Acta Geologica Sinica* **2**, 399–407 (2011).
5. Tong, J.-N., Huang, Y.-F. & Liang, L. Early Triassic biological-environmental-chronological stratigraphy. *Earth Science Frontiers* **21**, 144–156 (2014).
6. Zhao, L.-S., Tong, J.-N. & Zuo, J.-X. Lower Triassic Conodont Biostratigraphical Sequence at West Pingdingshan Section, Chaohu, Anhui Province, China. *Earth Science* **28**, 414–418 (2003).
7. Zhao, L.-S., Tong, J.-N., Michael, J. O. & Zuo, J.-X. Lower Triassic Conodont Zonations of Chaohu Area, Anhui Province and Their Global Correlation. *Earth Science* **20**, 623–634 (2005).
8. Zhao, L.-S. et al. Lower Triassic conodont sequence in Chaohu, Anhui Province, China and its global correlation. *Palaeogeography, Palaeoclimatology, Palaeoecology* **252**, 24–38 (2007).
9. Zhao, L.-S., Tong, J.-N., Sun, Z.-M. & Orchard, M. J. A detailed Lower Triassic conodont biostratigraphy and its implications for the GSSP candidate of the Induan–Olenekian boundary in Chaohu, Anhui Province. *Progress in Natural Science* **18**, 79–90 (2008).
10. Liang, D., Tong, J.-N. & Zhao, L.-S. Lower Triassic Smithian-Spathian Boundary at West Pingdingshan Section in Chaohu, Anhui Province. *Science China Earth*

Science **41**, 149–157 (2011).

11. Ji, C. et al. Ammonoid age control of the Early Triassic marine reptiles from Chaohu (South China). *Palaeoworld* **24**, 277–282 (2015).
12. Wang, G.-X. Marine Triassic of Anhui Province, Anhui Science & Technology Publishing House, 1–73 (1984).
13. Bureau of Geology and Mineral Resources of Anhui Province. Regional Geology of Anhui Province. Beijing (1987).
14. Motani, R., Jiang, D.-Y., Tintori, A., Rieppel, O. & Chen, G.-B. Terrestrial origin of viviparity in mesozoic marine reptiles indicated by early triassic embryonic fossils. *PLoS One* **9**, e88640 (2014).
15. Li, S.-Y., Tong, J.-N., Liu, K.-Y., Wang, F.-J. & Huo, Y.-Y. The Lower Triassic cyclic deposition in Chaohu, Anhui Province, China. *Palaeogeography, Palaeoclimatology, Palaeoecology* **252**, 188–199 (2007).
16. Jiang, D.-Y. et al. Biodiversity and sequence of the middle triassic panxian marine reptile fauna, guizhou province, china. *Acta Geologica Sinica* **83**, 451–459 (2009).
17. Zhang, Y.-Y., Jiang, D.-Y., Fu, W.-L., Ji, C. & Sun, Z.-Y. Microfacies characteristics of the Lower Triassic containing *Chaohusaurus* fauna in Chaohu area, Anhui Province and its palaeoenvironment. *Journal of Palaeogeography* **16**, 761–768 (2014).
18. Zhang, Y.-Y. et al. Microfacies and Palaeoenvironment analyses of the Middle-Upper member of the Nanlinghu formation (Lower Triassic), Chaohu, Anhui Province. *Journal of Stratigraphy* **40**, 290–296 (2016).
19. Sander, P. M. & Faber, C. The Triassic marine reptile *Omphalosaurus*: osteology, jaw anatomy, and evidence for ichthyosaurian affinities. *Journal of Vertebrate Paleontology* **23**, 799–816 (2003).
20. Chen, X.-H., Sander, P. M., Cheng, L. & Wang, X. A new Triassic primitive ichthyosaur from Yuanan, south China. *Acta Geologica Sinica* **87**, 672–677 (2013).
21. Chen, X.-H., Motani, R., Cheng, L., Jiang, D.-Y. & Rieppel, O. A carapace-like

bony “body tube” in an early Triassic marine reptile and the onset of marine tetrapod predation. *PLoS ONE* **9**, e94396 (2014a).

22. Chen, X.-H., Motani, R., Cheng, L., Jiang, D.-Y. & Rieppel, O. A small short-necked hupehsuchian from the lower Triassic of Hubei Province, China. *PLoS ONE* **9**, e115244 (2014b).
23. Motani, R. et al. A basal ichthyosauriform with a short snout from the Lower Triassic of China. *Nature* **517**, 485–488 (2015).
24. Wu, X. C., Zhao, L. J., Sato, T., Gu, S. X. & Jin, X. S. Anew specimen of *Hupehsuchus nanchangensis* Young, 1972 (Diapsida, Hupehsuchia) from the Triassic of Hubei, China. *Historical Biology* **28**, 43–52 (2016).
25. Jiang, D.-Y. et al. A large aberrant stem ichthyosauriform indicating early rise and demise of ichthyosauromorphs in the wake of the end-Permian extinction. *Scientific Reports* **6**, 1–9 (2016).
26. Zhou, M. et al. The cranial osteology revealed by three-dimensionally preserved skulls of the Early Triassic ichthyosauriform *Chaohusaurus chaoxianensis* (Reptilia: Ichthyosauromorpha) from Anhui, China. *Journal of Vertebrate Paleontology* **37**, e1343831 (2017).
27. Cheng, L., Motani, R., Jiang, D.-Y., Yan, C.-B., Tintori, A. & Rieppel, O. Early Triassic marine reptile representing the oldest record of unusually small eyes in reptiles indicating non-visual prey detection. *Scientific Reports* **9**, 1–11 (2019).
28. Huang, J.-D. et al. The new ichthyosauriform *Chaohusaurus brevifemoralis* (Reptilia, Ichthyosauromorpha) from Majiashan, Chaohu, Anhui Province, China. *PeerJ* **7**, e7561 (2019).
29. Ekeheien, C.P., Delsett, L. L., Roberts, A. J. & Hurum, J. H. Preliminary report on ichthyopterygian elements from the Early Triassic (Spathian) of Spitsbergen. *Norwegian Journal of Geology* **98**, 219–237 (2018).
30. Motani, R., Jiang, D.-Y., Tintori, A., Ji, C. & Huang, J.-D. Pre-versus post-mass extinction divergence of Mesozoic marine reptiles dictated by time-scale dependence of evolutionary rates. *Proceedings of the Royal Society B: Biological Sciences* **284**, 20170241 (2017).

31. Huang, J.-D. et al. Repeated evolution of durophagy during ichthyosaur radiation after mass extinction indicated by hidden dentition. *Scientific Reports* **10**, 1–10 (2020).

32. Merriam, J. C. Preliminary note on a new marine reptile from the Middle Triassic of Nevada. *Univ. Calif. Pubis., Bull. Dept. Geol.* **5**, 75–79 (1906).

33. Merriam, J. C. & Bryant, H. C. Notes on the dentition of *Omphalosaurus*. *University of California Publications, Bulletin of the Department of Geology* **6**, 329–332 (1911).

34. Sander, P. M. & Faber, C. New finds of *Omphalosaurus* and a review of Triassic ichthyosaur paleobiogeography. *Paläontologische Zeitschrift* **72**, 149–162 (1998).

35. Wintrich, T., Hagdorn, H. & Sander, P. M. An enigmatic marine reptile—the actual first record of *Omphalosaurus* in the Muschelkalk of the Germanic basin. *Journal of Vertebrate Paleontology* **37**, e1384739 (2017).

36. Mazin, J. M. & Bucher, H. *Omphalosaurus nettarhynchus*, une nouvelle espèce d`Omphalosauridé (Reptilia, Ichthyopterygia) du Spathien de la Humboldt Range (Nevada, U.S.A.). *Comptes-Rendus de l'Academie des Sciences, Paris* **305**, 823–828 (1987).

37. Tichy, G. Ein früher, durophager Ichthyosaurier (Omphalosauridae) aus der Mitteltrias der Alpen. *Geologisch-Paläontologische Mitteilungen Innsbruck* **20**, 349–369 (1995).

38. Wiman, C. Notes on the marine Triassic reptile fauna of Spitzbergen. *University of California publications, Bulletin of the department of geology* **10**, 63–73 (1916).

39. Carroll, R. L. & Dong, Z.-M. *Hupehsuchus*, an enigmatic aquatic reptile from the Triassic of China, and the problem of establishing relationships. *Philosophical Transactions of the Royal Society B: Biological Sciences* **331**, 131–153 (1991).

40. Motani, R. & You, H. Taxonomy and limb ontogeny of *Chaohusaurus geishanensis* (Ichthyosauria), with a note on the allometric equation. *Journal of Vertebrate Paleontology* **18**, 533–540 (1998).

41. Motani, R. Skull of *Grippia longirostris*: no contradiction with a Diapsid affinity

for the Ichthyopterygia. *Palaeontology* **43**, 1–14 (2000).

42. Cuthbertson, S. R., Russell, A. P. & Anderson, J. S. Cranial morphology and relationships of a new grippidian (Ichthyopterygia) from the Vega-Phroso Siltstone Member (Lower Triassic) of British Columbia, Canada. *Journal of Vertebrate Paleontology* **33**, 831–847 (2013).
43. Scheyer, T. M., Neenan, J. M., Bodogan, T., Furrer, H., Obrist, C. & Plamondon. M. A new, exceptionally preserved juvenile specimen of *Eusaurosphargis dalsassoi* (Diapsida) and implications for Mesozoic marine diapsid phylogeny. *Scientific reports* **7**, 1–22 (2017).
44. Nosotti, S. & Rieppel, O. *Eusaurosphargis dalsassoi* n. gen n. sp., a new, unusual diapsid reptile from the Middle Triassic of Besano (Lombardy, N Italy). *Memorie della Società Italiana di Scienze Naturali e del Museo Civico di Storia Naturale di Milano* **31**, 3–33 (2003).
45. Rieppel, O. & Lin, K. Pachypleurosaurs (Reptilia: Sauropterygia) from the Lower Muschelkalk, and a review of the Pachypleurosauroidea. (1995).
46. Rieppel, O. 'Sauropterygia I' in Wellnhofer P. (ed) Encyclopedia of Paleoherpetology, Volume 12A, Verlag Dr. Friedrich Pfeil, Munich, 1–134 (2000).
47. Klein, N. Skull morphology of *Anarosaurus heterodontus* (Reptilia: Sauropterygia: Pachypleurosauria) from the Lower Muschelkalk of the Germanic Basin (Winterswijk, The Netherlands). *Journal of Vertebrate Paleontology* **29**, 665–676 (2009).
48. Klein, N. Postcranial morphology and growth of the pachypleurosaur *Anarosaurus heterodontus* (Sauropterygia) from the Lower Muschelkalk of Winterswijk, The Netherlands. *Paläontologische Zeitschrift* **86**, 389–408 (2012).
49. Mann, A., McDaniel, E. J., McColville, E. R. & Maddin, H. C. *Carbonodraco lundi* gen et sp. nov., the oldest parareptile, from Linton, Ohio, and new insights into the early radiation of reptiles. *Royal Society Open Science* **6**, 191191 (2019).
50. deBraga, M. The postcranial skeleton, phylogenetic position, and probable lifestyle of the Early Triassic reptile *Procolophon trigoniceps*. *Canadian Journal*

of Earth Sciences **40**, 527–556 (2003).

51. Gow, C. E. The osteology and relationships of the Millerettidae (Reptilia: Cotylosauria). *Journal of Zoology* **167**, 219–264 (1972).
52. Modesto, S. P. The postcranial skeleton of the aquatic parareptile *Mesosaurus tenuidens* from the Gondwanan Permian. *Journal of Vertebrate Paleontology* **30**, 1378–1395 (2010).
53. Rieppel, O. *Helveticosaurus zollingeri* Peyer (Reptilia, Diapsida) skeletal paedomorphosis, functional anatomy and systematic affinities. *Palaeontographica, A* **208**, 123–152 (1989).
54. Jiang, D. Y., Motani, R., Hao, W. C., Rieppel, O., Sun, Y. L., Schmitz, L. & Sun, Z. Y. First record of Placodontoidea (Reptilia, Sauropterygia, Placodontia) from the eastern Tethys. *Journal of Vertebrate Paleontology* **28**, 904–908 (2008a).
55. Diedrich, C. G. Palaeoecology of *Placodus gigas* (Reptilia) and other placodontids—Middle Triassic macroalgae feeders in the Germanic Basin of central Europe—and evidence for convergent evolution with Sirenia. *Palaeogeography, Palaeoclimatology, Palaeoecology* **285**, 287–306 (2010).
56. Drevermann, F. R. Die Placodontier. 3. Das Skelett von *Placodus gigas* Agassiz im Senckenberg-Museum. *Abhandlungender senckenbergischen naturforschenden Gesellschaft* **38**, 319–364 (1933).
57. Li, C., Jiang, D. Y., Cheng, L., Wu, X. C. & Rieppel, O. A new species of *Largocephalosaurus* (Diapsida: Saurosphargidae), with implications for the morphological diversity and phylogeny of the group. *Geological Magazine* **151**, 100–120 (2014).
58. Li, C., Rieppel, O., Wu, X. C., Zhao, L. J. & Wang, L. T. A new Triassic marine reptile from southwestern China. *Journal of Vertebrate Paleontology* **31**, 303–312 (2011).
59. Hirasawa, T., Nagashima, H. & Kuratani, S. The endoskeletal origin of the turtle carapace. *Nature Communications* **4**, 1–7 (2013).
60. Jiang, D. Y., Rieppel, O., Motani, R., Hao, W. C., Sun, Y. L., Schmitz, L. & Sun, Z. Y. A new middle Triassic eosauropterygian (Reptilia, Sauropterygia) from

southwestern China. *Journal of Vertebrate Paleontology* **28**, 1055–1062 (2008b).

61. Wu, X. C., Cheng, Y. N., Li, C., Zhao, L. J. & Sato, T. New information on *Wumengosaurus delicatomandibularis* Jiang et al., 2008 (Diapsida: Sauropterygia), with a revision of the osteology and phylogeny of the taxon. *Journal of Vertebrate Paleontology* **31**, 70–83 (2011).
62. Rieppel, O. Osteology of *Simosaurus gaillardoti* and the relationships of stem-group Sauropterygia. *Fieldiana (Geology), ns* **28**, 1–85 (1994a).
63. Rieppel, O. The braincases of *Simosaurus* and *Nothosaurus*: monophyly of the Nothosauridae (Reptilia: Sauropterygia). *Journal of Vertebrate Paleontology* **14**, 9–23 (1994b).
64. Rieppel, O., Sander, P. M. & Storrs, G. W. The skull of the pistosaur *Augustasaurus* from the Middle Triassic of northwestern Nevada. *Journal of Vertebrate Paleontology* **22**, 577–592 (2002).
65. Sues, H. D. Postcranial skeleton of *Pistosaurus* and interrelationships of the Sauropterygia (Diapsida). *Zoological Journal of the Linnean Society* **90**, 109–131 (1987).
66. Sander, P. M., Rieppel, O. C. & Bucher, H. A new pistosaurid (Reptilia: Sauropterygia) from the Middle Triassic of Nevada and its implications for the origin of the plesiosaurs. *Journal of Vertebrate Paleontology* **17**, 526–533 (1997).
