# Supplementary material for: A globally distributed durophagous marine reptile clade supports the rapid recovery of pelagic ecosystems after the Permo-Triassic mass extinction
Source: Commun Biol. 2022 Nov 14;5:1242. doi: 10.1038/s42003-022-04162-6 (PMC9663502; doi:10.1038/s42003-022-04162-6)
Supplement: Supplementary file 2 — Description of Additional Supplementary Files [file 42003_2022_4162_MOESM2_ESM.pdf]

Description of Additional Supplementary Files

File name: Supplementary Data 1
Description: Character list and matrix for the relationships of diapsids.

File name: Supplementary Data 2
Description: The source data behind Figures 9 & S3 in the paper.

File name: Supplementary Data 3
Description: The source data behind Figure 10 in the paper.

File name: Supplementary Data 4
Description: The source data behind Figure 10 in the paper.
