# Supplementary material for: A globally distributed durophagous marine reptile clade supports the rapid recovery of pelagic ecosystems after the Permo-Triassic mass extinction
Source: Commun Biol. 2022 Nov 14;5:1242. doi: 10.1038/s42003-022-04162-6 (PMC9663502; doi:10.1038/s42003-022-04162-6)
Supplement: Supplementary file 3 — Supplementary Data 1 [file 42003_2022_4162_MOESM3_ESM.docx]

**Supplementary Data 1. Character list and matrix for the relationships of diapsids.**

The matrix was modified from the recent taxon-character matrix for diapsids with a particular focus on marine reptiles^25^. Two new characters were added to the original data matrix. The new specimen (HFUT MJS-16-012) was included in the matrix independently of the holotype of *Sclerocormus*, and two more taxa, *Eusaurosphargis* and *Omphalosaurus*, were included as well. The coding of *Eusaurosphargis* was updated based on ref.^43-44^. The coding of *Omphalosaurus* was based on the literature and direct observations. In addition, the OTU for Pachypleurosauria was replaced with an *Anarosaurus*-*Dactylosaurus* OUT^45-48^.

**Character Coding Revision**

**Parareptilia**

Character 142 (?→3) based on the absence of the median gastral element in *Carbonodraco lundi*^49^, *Procolophon trigoniceps*^50^, *Milleropsis* sp.^51^ and *Mesosaurus tenuidens*^52^.

***Helveticosaurus***

Character 154 (0→2) based on ref.^53^ fig. 6.

***Placodus***

Character 8 (1→0) based on ref.^46^ fig. 14.

Character 29 (0→1) based on ref.^46^ fig. 14.

Character 30 (1→0) based on ref.^46^ fig. 14.

Character 46 (0→1) based on ref.^46^ fig. 14.

Character 48 (0→?) based on ref.^46^ fig. 14. The absence of the quadratojugal results in this character being inapplicable for *Placodus*.

Character 51 (0→?) after ref.^46^. Absence of supratemporal in *Placodus* results in this character becoming inapplicable for *Placodus*.

Character 59 (1→0) based on ref.^46^ fig. 14.

Character 137 (1→2) based on ref.^54^ figs. 1, 3B.

Character 139 (0→1) based on ref.^55^ (pachyostotic gastral ribs).

Character 141 (?→0) based on ref.^56^ abbs. 6–7.

Character 158 (1→0) based on ref.^56^ taffs. 15–16.

Character 164 (1→2) based on ref.^54^ fig. 3.

***Largocephalosaurus***

Character 25 (0→1) based on ref.^57^ figs. 1B–C. 1A–B.

Character 31 (1→0) based on ref.^57^ figs. 1B–C. 1A–B.

Character 35 (3→1) based on ref.^57^ figs. 1B–C. 1A–B.

Character 43 (0→2) based on ref.^57^ figs. 1B–C. 1A–B.

Character 44 (2→0) based on ref.^57^ figs. 1B–C. 1A–B.

Character 46 (0→1) based on ref.^57^ figs. 1B–C. 1A–B.

Character 48 (1→?) as absence of quatradojugal causes this character to be inapplicable for *Largocephalosaurus*.

Character 49 (?→0) based on ref.^57^ figs. 1B–C. 1A–B.

Character 68 (?→1) based on ref.^57^ fig. 3A–B.

Character 71 (?→0) based on ref.^57^ fig. 3A–B.

Character 76 (2→0) after ref.^57^ fig. 3A–B.

Character 80 (0→?) as character state not possible to score unambiguously based on ref.^57^ fig. 3A–B.

Character 88 (0→1) based on ref.^57^ fig. 3A–B.

Character 90 (0→1) based on ref.^57^ fig. 1B–C, 3A–B.

Character 95 (0→?) because anterior extent of surangular indeterminate in ref.^57^ fig. 1B–C.

Character 111 (?→1) based on ref.^57^ fig. 1B–C.

Character 136 (?→0) based on ref.^57^ fig. 5D.

Character 141 (0→1) based on ref.^57^ fig. 4F.

Character 205 (2→1) following ref.^57^ fig. 6B.

Character 213 (0→1) following ref.^57^ fig. 6D.

***Sinosaurosphargis***

Character 24 (0→?) as upper temporal fenestrae are absent in *Sinosaurosphargis*, making this character inapplicable.

Character 25 (0→?) (inapplicable; see character 24).

Character 35 (3→1) based on ref.^58^ fig 2A, C.

Character 43 (0→2) based on ref.^58^ fig. 2.

Character 44 (2→0) based on ref.^58^ fig. 2A, C.

Character 46 (0→1) based on ref.^58^ fig. 2A, C.

Character 48 (1→?) as absence of quatradojugal causes this character to be inapplicable for *Sinosaurosphargis*.

Character 51 (0→?) after ref.^46^. Presence of supratemporal in *Sinosaurisphargis* uncertain ref.^57-58^ so character becomes inapplicable.

Character 75 (?→1) after ref.^58^ fig 2B, D.

Character 76 (2→0) after ref.^58^ fig. 2B, D.

Character 124 (2→1) after ref.^58^ fig. 3A–B.

Character 130 (0→?) as sacral vertebrae/ribs not preserved in *Sinosaurosphargis*^58^.

Character 158 (?→0) based on personal observation of unpublished specimen on display at Shanghai Museum of Natural History.

Character 175 (?→0) based on ref.^59^ fig. 4B.

Character 202 (1→?) because coracoid foramen not discernible in any of the known *Sinosaurosphargis* specimens^58^.

Character 205 (2→?) (detailed humerus morphology unknown in *Sinosaurosphargis*).

Character 206 (?→1) as presence of thyroid fenestra can be inferred from morphology of preserved pelvic girdle bones in ref.^59^ fig. 4B.

Character 207 (4→0) following ref.^58^ fig. 2A–B.

Character 208 (0→?) as sacral and caudal region unknown for *Sinosaurosphargis*^58^.

Character 209 (1→?) (detailed humerus morphology unknown in *Sinosaurosphargis*).

Character 210 (?→1) following ref.^59^ fig. 4B.

Character 213 (1→?) as hindlimb unknown for *Sinosaurosphargis*^58^.

Character 216 (?→0) based on ref.^58^ fig. A–B.

Character 218 (?→0) based on personal observation of unpublished specimen on display at Shanghai Museum of Natural History.

***Wumengosaurus***

Character 6 (1→?) because none of the preserved skulls are exposed in direct dorsal or ventral view, not allowing this character to be scored unambiguously^60-61^.

Character 7 (1→0) after ref.^60^ fig. 3 and ref.^61^ fig. 2A–B.

Character 13 (1→0) after ref.^61^ fig. 2A–B, D.

Character 15 (0→?) as the lacrimal in *Wumengosaurus* forms a complex with the prefrontal ref.^61^ figs 2A–B, D and 3A, E that is morphologically and topologically similar to the prefrontal in some pachypleurosaurs (e.g. ref.^46^ fig. 31) and is possibly homologous to it.

Character 16 (1→?) due to the uncertainty regarding the presence or absence of the lacrimal in *Wumengosaurus* (see character 15).

Character 18 (0→1) based on ref.^61^ fig. 2D.

Character 23 (0→?) based on the uncertain identity of the lacrimal (see characters 15–16).

Character 25 (0→1) based on ref.^61^ fig. 2D.

Character 27 (0→?) (inapplicable) as frontal-nasal suture is V-shaped in *Wumengosaurus*, with frontal forming anterior and anterolateral prongs ref.^61^ fig. 2D.

Character 28 (?→0) based on ref.^61^ fig. 2D.

Character 29 ((01)→1) on the basis of the best preserved skull roof ref.^61^ fig. 2D.

Character 35 (?→0) based on ref.^61^ fig. 2D.

Character 36 (?→0) after ref.^61^ fig. 2D.

Character 69 (?→1) after ref.^61^.

Character 74 (?→0) following ref.^61^ fig. 3A, E.

Character 76 (?→1) after ref.^61^ fig. 3A, E.

Character 77 (?→1) after ref.^61^ fig. 3A, E.

Character 81 (0→2) after ref.^61^ fig. 3A, E.

Character 83 (?→1) following ref.^61^ fig. 3A, E.

Character 103 (1→(0 1)) after ref.^61^ fig. 2A–B, D and 3A, E.

Character 114 (0→1) after ref.^61^.

Character 120 (1→0) following ref.^61^.

Character 129 (0→1) based on ref.^61^ fig. 1F.

Character 131 (?→0) following ref.^61^ fig. 1F.

Character 135 (?→0) following ref.^60^ figs 1–2, 4 and ref.^61^ fig. 1A–C.

Character 139 (0→1) based on ref.^61^.

Character 144 (0→?) character state difficult to determine unambiguously as available specimens preserved in lateral, dorsal, dorsolateral and ventrolateral views ref.^60^ figs 1–2 and ref.^61^ fig. 1A–C.

Character 147 (1→?) because lack of fully articulated pectoral girdles hinders scoring this character unambiguously ref.^60^ fig. 4 and ref.^61^ fig 4C–D.

Character 149 (0→2) following ref.^61^ fig. 4D.

Character 157 (0→1) following ref.^61^ fig. 4D.

Character 159 (0→(0 1)) following ref.^60^ fig. 4 and ref.^61^ fig. 5A–C.

Character 162 (3→(0 3)) after ref.^60^ fig. 4H and ref.^61^ fig. 5A–C.

Character 178 (1→(0 1)) following ref.^60^ figs 5–6 and ref.^61^ fig. 5D–E.

Character 181 (?→0) based on ref.^61^ fig. 6 and ref.^61^ figs 5D–E.

Character 182 (0→2) after ref.^60^ fig. 6 and ref.^61^ figs 5D–E.

Character 184 (?→1) after ref.^60^ fig. 6 and ref.^61^ figs 5D–E.

Character 185 (?→1) after ref.^60^ fig. 6 and ref.^61^ figs 5D–E.

Character 190 (?→0) following ref.^61^ fig. 2D.

Character 193 (3→2) after ref.^61^ fig. 4A–B.

Character 205 (0→1) following ref.^60^ fig. 4 and ref.^61^ fig. 5A–C.

Character 211 (?→2) after ref.^60^ fig. 6 and ref.^61^ figs 5D–E.

Character 213 (2→1) after ref.^60^ fig. 6 and ref.^61^ figs 5D–E.

***Simosaurus***

Character 130 (1→0) based on ref.^62^ fig. 9.

Character 36 (1→2) based on ref.^62^ fig. 9.

Character 38 (0→1) based on ref.^62^ fig. 9.

Character 67 (?→1) based on ref.^63^ fig. 3A.

Character 164 (1→2) based on ref.^62^ fig. 30A.

**Pistosauridae**

Character 30 (1→0) based on *Augustasaurus hagdorni* in ref.^64^ fig. 2.

Character 139 ((0 1)→0) based on the absence of pachyostotic ribs in *Pistosaurus*^65^ and *Augustasaurus*^66^.

***Sclerocormus parviceps***

Character 4 (?→0) based on the direct observation.

Character 113 (0→?) based on the direct observation.

***Cartorhynchus***

Character 28 ((0 1)→0) based on the recent restudy^31^.

Character 100 (?→0) based on the recent restudy^31^.

Character 101 (?→1) based on the recent restudy^31^.

Character 105 (?→1) based on the recent restudy^31^.

Character 106 (?→0) based on the recent restudy^31^.

Character 108 (?→1) based on the recent restudy^31^.

Character 109 (?→1) based on the recent restudy^31^.

Character 110 (?→2) based on the recent restudy^31^.

Character 111 (?→1) based on the recent restudy^31^.

Character 112 (?→0) based on the recent restudy^31^.

Character 113 (0→1) based on the recent restudy^31^.

Character 114 (?→0) based on the recent restudy^31^.

**Two New Characters**

219. Dental battery: (0) absent; (1) present; (2) dentary edentulous

220. Orange peel-like enamel surface: (0) absent; (1) present

**Character Description**

1. Preorbital and postorbital regions of skull: (0) of subequal length; (1) preorbital region distinctly longer than postorbital region; (2) postorbital region distinctly longer

2. Premaxilla postnarial process, restricting the contact of the maxilla to the external nares even excluding maxilla: (0) without; (1) with

3. Premaxillae: (0) enter internal naris; (1) excluded

4. Shape of premaxilla: (0) Horizontal ventral margin; (1) down-turned ventral margin

5. Form of suture between premaxilla and maxilla above dentigerous margin: (0) Simple vertical; (1) diagonal contact; (2) notch present in maxilla

6. Snout: (0) unconstructed; (1) constricted

7. Maxilla ascending process between naris and orbit: (0) absent; (1) present

8. Maxilla extends to posterior orbital margin: (0) TRUE; (1) FALSE

9. Maxilla orbital exposure: (0) absent; (1) present

10. Nasals: (0) shorter; (1) longer than frontals

11. Nasals: (0) meet in dorsomedial suture; (1) separated from one another by nasal processes of the premaxillae extending back to the frontal bone(s)

12. Nasals: (0) paired; (1) fused; (2) lost

13. Nasal, extends anteriorly much beyond exn: (0) false; (1) true

14. Lacrimal duct: (0) enclosed by lacrimal only; (1) lateral border formed by maxilla

15. Lacrimal: (0) enters the orbital margin; (1) remains excluded therefrom due to an extemal contact between the posteroventral part of the prefrontal and the posterodorsal margin of the maxilla

16. The lacrimal: (0) present and enters the external naris; (1) present but remains excluded from the external naris by a contact of maxilla and nasal; (2) absent

17. External nares: (0) Separate; (1) single, medial naris

18. External nares location: (0) Marginal; (1) close to midline

19. External nares shape: (0) Rounded; (1) elongate

20. Septomaxilla: (0) present; (1) absent

21. The prefrontal and postfrontal: (0) separated by the frontal along the dorsal margin of the orbit; (1) a contact of prefrontal and postfrontal excludes the frontal from the dorsal margin of the orbit

22. Prefrontal palatine antorbital contact: (0) narrow forming less than one third the transverse distance between the orbits; (1) forming at least one half the distance

23. Prefrontal and maxilla: (0) do not meet due to a contact of lacrimal and nasal; (1) prefrontal contacts maxilla anteriorly and thereby separates lacrimal and nasal from one another

24. Upper temporal fenestra: (0) Oval in outline and not elongated caudally; (1) elongated caudally with inner surface of parietal and squamosal facing dorsally

25. Frontal: (0) widely separated from the upper temporal fossa; (1) narrowly approaches or; (2) enters the anteromcdial margin of the upper temporal fossa

26. Frontal(s) distinct posterolateral processes: (0) without; (1) with

27. Frontal anterior margins: (0) frontal suture with nasal transverse; (1) oblique forming an angle of at least 30° with long axis of the skull

28. Frontal proportions: (0) length exceeds width by at least four times; (1) length no greater than twice the width

29. frontal, butterfly-shaped with antero- and postero-lateral processes: (0) false; (1) true

30. Postorbital and parietal contact: (0) absent; (1) present

31. Postorbital posterior extent: (0) terminates prior to reaching posterior limit of parietal; (1) extends to at least the posterior limit of the parietal

32. Postorbital: posterior process contacting squamosal: (0) long; (1) short

33. The postorbital: (0) in contact with the supratemporal; (1) not in contact

34. Postfrontal: (0) excluded from upper temporal fenestra; (1) enters upper temporal fenestra

35. Pineal foramen: (0) close to the middle of the skull table; (1) displaced anteriorly; (2) absent

36. Parietal skull table: (0) broad; (1) weakly constricted; (2) strongly constricted (at least posteriorly); (3) forms a sagittal crest

37. Parietal shelf for adductor musculature: (0) absent; (1) present as shallow excavations on the lateral parietal margin

38. Skull roof distinct posterior emargination: (0) absent; (1) present

39. Postparietals: (0) present; (1) absent

40. Tabulars: (0) present; (1) absent

41. The jugal: (0) no farther than to the middle of the cheek region; (1) nearly to the posterior end of the skull

42. Subtemporal process of jugal: (0) robust with height >50 % of length; (1) slender with height <50 % of length; (2) absent

43. Lower temporal fossa: (0) absent; (1) present and closed ventrally; (2) present but open ventrally

44. Squamosal: (0) descends to ventral margin of skull; (1) reaches only the approximate mid level of the lower temporal fossa; (2) remains distinctly restricted to the dorsal region of the cheek

45. Squamosal Occipital flange: (0) absent or poorly developed forming only a thin ridge; (1) well developed forming a broadly exposed lappet

46. Quadratojugal: (0) present; (1) absent

47. Quadratojugal: (0) remains restricted to the ventral margin of the cheek; (1) shows distinct dorsal extension

48. Quadratojugal anterior process: (0) present; (1) absent

49. Supratemporals: (0) present; (1) absent

50. Supratemporal: (0) stout and short; (1) long and slender, projecting far anteriorly; (2) same as 1 but also contacting the frontal

51. supratemporal occipital lappet: (0) absent; (1) present

52. Exoccipitals meet dorsal to the basioccipital condyle: (0) TRUE; (1) FALSE

53. Supraoccipital: (0) exposed more or less vertically on occiput; (1) more or; (2) less horizontally at posterior end of parietal skull table

54. Occipital crest: (0) absent; (1) present

55. Paroccipital process: (0) ends freely; (1) reaches suspensorium

56. Paroccipital processes: (0) extend laterally forming 90° with parasagittal plane; (1) deflected posterolaterally at an angle of about 20° from the transverse width of the skull; (2) deflected dorsolaterally at an angle of nealry 45°

57. Paroccipital processes: (0) slender; (1) heavy with anteroposterior dimension at least one third greater than dorsoventral dimension

58. Quadrate: (0) has straight posterior margin; (1) the quadrate shaft deeply excavated (concave) posteriorly

59. Quadrate: (0) covered by squamosal and quadratojugal in lateral view; (1) exposed in lateral view

60. Quadrate anterior process: (0) long, extending forward along its sutural contact with the quadrate process of the pterygoid to nearly reach the level of the transverse flange; (1) short, not extending anteriorly beyond 55% the length of the quadrate process of the pterygoid

61. Lateral conch on quadrate: (0) absent; (1) present

62. Prootic / parietal contact: (0) absent; (1) present

63. Stapes morphology: (0) robust with its greatest depth exceeding one third of its total length; (1) slender with the length at least four times the depth

64. Stapedial dorsal process; (0) present as ossified process; (1) absent

65. Medial wall of inner ear: (0) unossified; (1) ossified

66. Basi / parasphenoid ratio: (0) narrowest transverse width no more than 60% of the maximum length measured from basipterygoid process to posteriormost limit; (1) narrowest part (waist) exceeds 80% of the length

67. Ventral braincase tubera: (0) absent; (1) present and restricted to baSioccipital; (2) present, very large, and restricted to basisphenoid

68. Basioccipital/basisphenoid relationship: (0) floor of braincase with gap between both elements; (1) elements fused to floor brain cavity

69. Sphenethmoid: (0) present; (1) absent

70. Dorsal wing of epipterygoid: (0) broad; (1) narrow

71. Orientation of basipterygoid processes: (0) anterolateral; (1) lateral

72. Palate: (0) kinetic; (1) akinetic

73. Choana palatal exposure: (0) parallel to medial border of maxilla; (1) deflected posteromedially; (2) hidden in palatal view

74. Suborbital fenestra: (0) absent; (1) present

75. Palatal process of pterygoid: (0) extends anterior to the anterior limit of the palatine; (1) forms oblique suture with palatine but process ends before reaching anterior limit of palatine; (2) forms transverse suture with palatine

76. Pterygoid flanges: (0) well developed; (1) strongly reduced

77. Pterygoid transverse flange: (0) extends ventrally below the level of the maxillary tooth row; (1) not

78. Transverse flange lateral margin: (0) posterolateral margin forms sharp edge with anteromedial margin; (1) posterolateral margin merges smoothly into anteromedial margin forming a smoothly convex lateral outline

79. Orientation of transverse flange of pterygoid: (0) directed predominantly laterally; (1) anterolaterally

80. Pterygoids: (0) in contact anteriorly; (1) separated anteriorly

81. Interpterygoid vacuity: (0) anterior end tapers sharply; (1) anterior border crescentic; (2) absent

82. Cultriform process: (0) long exceeding length of parasphenoid body and reaching forward to the level of the posterior limit of the internal nares; (1) short not reaching the level of the internal nares

83. Ectopterygoid: (0) present; (1) absent

84. Contact between ectopterygoid and jugal: (0) No contact or; (1) restricted area of contact approximately equal to or; (2) less than contact between ectopterygoid and pterygoid; (3) ectopterygoid expanded caudally

85. Contact between ectopterygoid and maxilla: (0) present; (1) absent

86. Shape of ectopterygoid along suture with pterygoid: (0) Transversely broad; (1) posteroventrally elongate and does not reach lateral corner of transverse flange; (2) posteroventrally elongate and reaches corner of transverse flange

87. Mandibular articulations: (0) approximately on a level with occipital condyle; (1) displaced to a level distinctly behind occipital condyle; (2) positioned anterior to the occipital condyle

88. Splenial bone: (0) enters the mandibular symphysis; (1) remains excluded therefrom

89. Dentary symphysis; (0) straight; (1) recurved

90. Retroarticular process of 1ower jaw: (0) absent; (1) present

91. Upturned retroarticular process: (0) absent; (1) present

92. Distinct dorsal process of lower jaw formed by the coronoid only: (0) absent; (1) present

93. Two or more coronoids: (0) present; (1) absent

94. Meckelian fossa faces: (0) mediodorsally; (1) dorsally due to greatly expanded prearticular

95. Surangular: (0) extends anterior to coronoid eminence; (1) terminates prior to reaching eminence

96. Surangular lateral shelf: (0) absent; (1) present

97. Angular lateral exposure: (0) exposed along one third the lateral face of the mandible; (1) exposed only as a small sliver along the lateral face; (2) absent from lateral aspect

98. Angular maximum exposed height relative to surangular: (0) subequal or angular deeper; (1) angular clearly shallower

99. Prearticular: (0) extends anterior to coronoid eminence; (1) terminates prior reaching eminence

100. Tooth implantation: (0) subthecodont; (1) thecodont; (2) ankylothecodont; (3) attached to the surface

101. One or two caniniform teeth on maxilla: (0) present; (1) absent

102. The contact between vomer and maxilla: (0) absent; (1) present

103. Large scleral ring filling the orbit: (0) absent; (1) present

104. Premaxilla dentition: (0) 4 or more; (1) 3 or less; (2) edentulous

105. Anterior (premaxillary and dentary) teeth: (0) upright; (1) strongly procumbent

106. The maxillary tooth row: (0) restricted to a level in front of the posterior margin of the orbit; (1) it extends backward

107. Diastema between maxillary and premaxillary teeth: (0) absent; (1) present

108. Parasphenoid teeth: (0) present; (1) absent

109. Palatine teeth: (0) present; (1) absent

110. Dentition on transverse flange of pterygoid: (0) present as shagreen of teeth; (1) present but with one large distinct row of teeth along the posterior edge of the transverse flange; (2) edentulous

111. Teeth on palatine ramus of pterygoid: (0) present; (1) absent

112. Heavy, conical teeth on vomer: (0) absent; (1) present

113. Button-like, durophagous teeth: (0) absent; (1) present

114. Marginal teeth with: (0) convex; (1) concave lingual surface of crown

115. Vertebrae; (0) notochordal; (1) nonnotochordal

116. Vertebrae: (0) amphicoelous; (1) platycoelous; (2) other

117. Neck: (0) short, with 5 or less vertebrae; (1) long with more than 5 vertebrae

118. Ratio of length of centra of mid-cervical and mid-dorsal vertebrae: (0) 1.0>=; (1) >1.0; (2) >1.5

119. Dorsal intercentra: (0) present; (1) absent

120. Cervical intercentra: (0) present; (1) absent

121. Zygosphene-zygantrum articulation: (0) absent; (1) present

122. Neural canal: (0) evenly proportioned; (1) distinctly higher than wide; (2) wider than high in Saurosphargis and Sinosaurosphargis

123. Sutural facets receiving the pedicels of the neural arch on the dorsal surface of the centrum in the dorsal region: (0) narrow; (1) expanded into a cruciform; (2) butterfly-shaped platform

124. Transverse processes of neural arches of the dorsal region: (0) relatively short; (1) distinctly elongated; (2) transverse process absent

125. Ratio of lengths of caudal transverse processes and centra: (0) >1.0; (1) 1.0>=

126. Caudal lateral projections (transverse processes) beyond fifth caudal: (0) absent; (1) present

127. Strong caudal neural spine anticlination: (0) absent; (1) present

128. Pre-and postzygapophyses do not show an anteroposterior trend of increasing inclination within the dorsal and sacral region: (0) TRUE; (1) FALSE

129. Cervical ribs, a distinct free anterior process: (0) absent; (1) present

130. The number of sacral ribs: (0) two; (1) three; (2) four or more

131. Slender and tapering cervical ribs at low angle to vertebrae: (0) absent; (1) present

132. Neural arches of mid-dorsals: (0) shallowly excavated; (1) deeply excavated

133. Most trunk ribs: (0) dichocephalous; (1) holocephalous but not clearly articulating with the neural arch only; (2) holocephalous and only articulating with the neural arch

134. Dorsal ribs: (0) without; (1) with distinct, fan-shaped uncinate process on the convex margin; (2) on the concave margin; (3) with a distinct crest on the dorsal surface of the shoulder region

135. Dorsal ribs: (0) slender; (1) dorsal ribs transversely broadened and in antero-posterior contact with each other, forming closed rib-basket

136. Second sacral rib: (0) not bifurcate; (1) bifurcate

137. Distal width of haemal spine: (0) equivalent to proximal width; (1) tapering; (2) wider than proximal width

138. Atlantal ribs: (0) ossified; (1) not ossified

139. Rib, pachyostosis: (0) absent; (1) present

140. Gastralia; (0) present; (1) absent

141. Lateral gastralia, expanded and flat: (0) false; (1) true

142. Median gastral element angulated: (0) angled anteriorly straight; (1) angled posterirorly absent

143. Osteoderms: (0) absent; (1) present; (2) present, dense and in sutural contact, providing closed dorsal body cover

144. Body compression: (0) semi-round to slightly compressed dorsoventrally; (1) strongly flattend dorso-ventrally; (2) bilaterally compressed

145. Cleithrum; (0) present; (1) absent

146. Clavicles medially: (0) broad; (1) narrow

147. Clavicles to the interclavicle: (0) positioned anteroventrally; (1) dorsally

148. Clavicle surface of scapula: (0) applied to the anterior (lateral); (1) to the medial

149. Posterior process on interclavicle: (0) elongate; (1) short; (2) rudimentary or absent

150. Supraglenoid buttress: (0) present; (1) absent

151. Cranial margin of interclavicle: (0) lacks a notch; (1) has distinct notch

152. Caudal stem of interclavicle expansion: (0) lascks distnctive expansion; (1) has distinct expansion

153. Mineralized sternum: (0) absent; (1) present

154. Scapula: (0) represented by a broad blade of bone; (1) slender, high, and narrow; (2) with a constriction separating a ventral glenoidal portion from a posteriorly directed dorsal wing

155. Coracoid process: (0) small; (1) large

156. Coracoid ossifications: (0) one; (1) two

157. Interclavicle anterior process or triangle: (0) conspicuously present; (1) absent

158. Pectoral fenestration: (0) absent; (1) present

159. Humerus: (0) rather straight; (1) "curved"

160. Humerus, anterior flange: (0) absent; (1) present

161. Humeral distal articulations: (0) distinct trochlea and capitellum; (1) low double condyle

162. The ectepicondylar groove: (0) open and notched anteriorly; (1) open without anterior notch; (2) closed (i.e., ectepicondylar foramen present) absent

163. Entepicondylar foramen: (0) present; (1) absent

164. Supinator process: large angled away irom humeral shaft: (0) large, angled away from humeral shaft; (1) large, confluent with shaft; (2) small or absent

165. Radius: (0) shorter than ulna; (1) longer than ulna; (2) approximately the same length

166. Radius, anterior flange: (0) absent; (1) present

167. Distal end of ulna: (0) not; (1) distinctly expanded (Sander et al., 1997 the short ulna of plesiosaurs with a convex postaxial margin is here treated as not comparable [?])

168. Olecranon: (0) well developed; (1) small or absent

169. Ulnare: (0) wider than long; (1) longer than wide; (2) as long as wide

170. Radiale larger than other capals: (0) false; (1) true

171. Metacarpal IV: (0) longer than metacarpal III; (1) equal or shorter

172. Forelimb digits: (0) at least some divergent; (1) all convergent

173. Dorsal margin of ilium anterior process: (0) absent; (1) present

174. Pubic tubercle: (0) if present small and and directed anteroventrally; (1) large and strongly turned ventrally tl

175. Ischium: (0) a broad, kidney-shaped plate; (1) with concave posterior and anterior margins

176. Acetabulum: (0) oval; (1) circular

177. Femur, distal and proximal extremities: (0) subequal widths; (1) distal one much wider

178. Femoral shaft: (0) stout and straight; (1) slender and sigmoidally curved

179. Anterior femoral condyle relative to posterior condyle: (0) larger and extends further distally; (1) smaller/equisized and of subequal extent distally

180. Fibula: (0) bowed away from tibia; (1) straight and not bowed away

181. Tibia/astragalus articulation: (0) loose fitting; (1) tightly fitting with well developed articulation

182. Astragalus/ distal tarsal IV articulation: (0) articulation poorly defined; (1) well defined; (2) absent

183. Calcaneal tuber: (0) absent; (1) present

184. Distal tarsal 1: (0) present; (1) absent

185. Distal tarsal 5: (0) present; (1) absent

186. Metatarsal 5: (0) long and slender; (1) distinctly shorter than the other metatarsals and with a broad base

187. Metatarsal 5: (0) straight; (1) hooked

188. Metatarsal V plantal tubercle: (0) absent; (1) present

189. Metatarsal 1/ IV ratio: (0) 1 greater than 50% the length of IV; (1) I less than 50 % the length of IV

190. Parietal(s) in the adult: (0) paired; (1) fused in their posterior part only; (2) fully fused

191. Distal femoral condyles: (0) prominent; (1) do not project markedly beyond shaft

192. Total number of tarsal ossifications: (0) four or more; (1) less than four

193. Proximal caudal neural spine height: (0) Moderately tall with heightllength > l.0 and <2.0; (1) low with height/length < 1.0; (2) tall with heightllength >2.0 and <3.0; (3) very tall with heightllength >3.0

194. Total number of carpal ossifications: (0) more than 3; (1) three; (2) two

195. Premaxilla longer than maxilla: (0) false; (1) true

196. Premaxillae: (0) small; (1) large

197. Nares: (0) positioned anteriorly; (1) situated in the central or posterior area of the antorbital skull portion

198. Humeral torsion: proximal and distal ends of humerus: (0) set off at 45° angles from one another; (1) angle between opposing ends reduced to not more than 20°

199. Dorsal count: (0) 19 or more; (1) 18 or less

200. Foramen for the supposed passage of the pes artery: (0) present; (1) absent between astragalus and calcaneum

201. Astragalus and calcaneum: (0) never fused in adult; (1) fused; (2) hinge present

202. Coracoid foramen: (0) enclosed by coracoid ossification; (1) lies between coracoid and scapula

203. Iliac blade: (0) well developed; (1) reduced

204. Femur, internal trochanter: (0) well-developed; (1) reduced

205. Deltopectoral crest: (0) well developed; (1) reduced

206. Thyroid fenestra: (0) absent; (1) present

207. Upper temporal fossae: (0) absent; (1) present and subequal in size; (2) slightly larger than the orbit; (3) present and distinctly larger than the orbit; (4) present and distinctly smaller than the orbit

208. Sacral (and caudal) ribs of transverse processes to their respective centrum: (0) sutured; (1) fused

209. Insertional crest for latissimus dorsi muscle: (0) prominent; (1) reduced

210. Pubis, processus lateralis: (0) present; (1) absent

211. Number of pedal centralia: (0) both lateral and medial centralia present; (1) medial pedal centrale lost; (2) both centralia lost

212. Humerus epicondyles: (0) prominent; (1) reduced

213. Intertrochanteric fossa: (0) deep; (1) distinct but reduced; (2) rudimentary or absent

214. Nasal, reaching the tip of snout: (0) false; (1) true

215. Flat gastral elements with caudad one outlying the craniad one: (0) absent; (1) present

216. Pineal foramen position relative to orbit: (0) posterior; (1) between orbits

217. Rostral and caudal median ridges on neural spines: (0) absent; (1) present

218. Carpus elongated, as long as the more distal elements or longer: (0) false; (1) true

219. Dental battery: (0) absent; (1) present; (2) dentary edentulous

220. Orange peel-like enamel surface: (0) absent; (1) present

**Character matrix for the relationships of diapsids (Fig. 9; Supplementary Fig. 3)**

Seymouriidae

00000000000000000000100??00000010?0000000?0000000000000000000000000000000000000000000000000000000?00000001010(01)11000000000000100000000(01)1000?0??000000000000010000000000000000000000000000000000000000000000000000000000000?00

Synapsida

(01)0000000(01)(01)0000000000000??000011(01)0?100(01)0000101000(01)0000000000000000000000000000000000000000(01)0000(01)0000000000(01)01(01)100000010000000100000010?0000?0??00000000000(01)010000000000001000(01)0000000000000000000(02)00000(01)000000000000000000000

Parareptilia

(01)00000(01)10000000(01)0000(01)1(01)??(01)0(01)010(01)0?(01)000000?(01)(01)10000000000000000000(01)(01)(01)(01)000001(01)00(01)(01)0(01)(01)00002(01)010(01)1(01)(01)(01)00(01)0(01)00000010{01}000000(01)000000011000(01)000?(02)000?0?3(012)(01)00000?000000(01)0000(012)0(12)000(01)(01)000(01)00(01)0010000000000000(01)00000(01)01000000000(12)000000{01}00

Captorhinidae

00010001000000000000000??00001011?(01)000010?001000000?0000000100000000000001000000001???0000?0100000000100000100000000000000?01000000000000001?000?000001000000000000200000000000000000000000000002000000000000000000000000000

Araeoscelidia

1000000100000?0000000000010001011000000000(01)010000001000000010?00?000??0001000010000010010000100000000000000000000000110000?01000000100001001?0000000001010101000000(12)00001000000000000000000010001000000000000030001000000000

Rhynchocephalia

0(02)000010100001?(12)000001100(01)0001(01)011(01)(02)1011(01)1(12)010(01)1(01)0010(01)1(01)0111(01)111001(01)1100011010000100(01)2010101101011131(01)00000102(01)000(01)010(01)0100011000000100(01)(12)100?00011100(01)0010001000(01)20100001000(01)0110111100(01)1111(01)(01)00(01)00000111000011100(01)010000000

Squamata

(012)0(01)000(01)0(01)00(012)010(12)0000(01)11000000(01)0(01)1(01)(012)(02)10110(12)2201??(01)(01)0(01)(01)(01)1001111111(01)01111(01)001(01)0011(01)010(01)01(012)101011(01)0(01)11(01)311000001(01)2(01)0001(02)1010(01)000110(01)00001000(12)101?0(02)01(01)10(01)10010101000(01)(02)11000(01)(01)010(01)011011(01)110111111(01)00100001(01)110000111002010000000

*Prolacerta*

1100000101000?0101100000011000011000111101200011000100110111011100111?0001001000000?01010110101001101000000101000010120100?0110010111?012?00??00110001100000000010122001101011010111001011101000200000?020000031001010000?00

*Trilophosaurus*

110000???0000???00111??1101000???1231111010??0????0?0?11111?01?110111000?100111?0100??2101011100??101002?0?11210001(12)100100?01??0101110000100?0??110001?1?000100?10020?00100011010111001011101000100000?020000011?110100?0000

Rhynchosauria

0111101000000?01111101101000001111231111112100111000001111110011?001110011001000010101010111101111121102001102000010110100?00100101110012100?0?0110001110010100010110001100011010111101011101100300000112?0000110010100?0010

Archosauriformes

(01)1(01)(01)101(01)(01)1000(01)0101110000(01)(01)00001010(02)(02)1?(01)1011(12)0010(01)0010(01)1(01)111?011111?11?1(01)0110100001(01)1(01)1(012)1011(01)111001?1110000(01)10(12)00001(012)10(01)(01)0(01)01010(01)1(02)112001(12)?00?0(12)01100011011001000(01)0110001(012)01011010111101(01)11100(01)(01)02000001(01)2(01)00001(01)0(01)1(01)(12)00?0000

*Claudiosaurus*

100000?110000?11010?0?0001?001101100101102201001100?001000?10????10???00010010100000120100001?10010010000001010000?0110000?01000101010000100000011000100?0001000110200010010000101100000000000101000001000001011101110000000

*Coelurosauravus*

00?0001111000???000???100000010011?011110221?0010?0????1?1110?11????1????????????????????1?01??????31?0?00??????0??010110?00?0?0001000?01??1??00????????0?????0?00020?002?0?00???101000000000010100100100?0000110010?000??00

Kuehneosauridae

0100001010000011110?00000100010010201011022200011301010001111???001??1000110?0010?00??0?0110100011?010000000010?0010101100?111?1?0002000??000000111000000100100?02120?00????00?10211001??100?01?10000???0000011100?1000?0000

*Acerosodontosaurus*

1???0??10????00?????0??00??0???0?1??????002001???????????0??0?????????????????????0????????11?????1?1????0??????0?00?00?0??0???0?0?01??????0????0?????????????00100?20?10?1?????????????????????0?????????0???3????0??0???00

*Tangasaurus*

?????????????????????????????????????????????????????????????????????????????????0??????????????????????????????????0??????0?????0??1???2???????????????1??0??0?100???????00???????????01???????0????01?????0???0??0????00??

*Youngina*

1000000000000001000?00?001?001101100??000110?000000100???0?10?1???????????10??00??00100?01??????00??10000001010000000?0?0000?10??0?01001??????00????0?1010?01?00100??0??0??0001??1??????000?10?0000000(01)???1??03????0?0?00000

*Thadeosaurus*

???????????????????????????????????????????????????????????????????????????0???????????????????????????????1?10????0?00?0??0??0?00?01001??000??0????0??010?01000120?20010000000?011100??0?0?1?1000???00?000000?10?100?0?00??

*Lanthanolania*

10???0?00?000?01????0?0????001?1????????012???????0????????????????????????0??????0?????????1?????????0????0?2?????????????????????????????????????????????????????????????????????????????????????0??????????3??????0????00

*Orovenator*

10?0000?100000000010000?010001???0{02}101???1{12}?????0?0??????0?10?00?0????000100000?000000?10??0?00001?01?000000000?0?00?????????????????????????????????????????????????????????????????????????0????000?????????3??????0????00

*Sophineta*

000000101000?101000?11102000?01?11001?11022000010????????1111???????????01?0?10???0000010??0???????310?0000???0?0?00?1??1?0010?0???01????????????????????????????????????????0?????????????000?????00????????010???????0??00

*Pamelina*

010000101000??11110000000101?10?1020101102220?0113???????1111????????11?01?0??0???00??0101?0?0?????010?00?0?010?0?10?0??0?0101?11??02????????????????????????????????????????????????????????0??1??00?????????11??????????00

*Tanystropheus*

11000011?1000?01011?00100010010111101111012201??0001001101110111?1111?100100100000000201010010100110100000011210001012110(012)?01100101020000?00?0001100011000000000101210012010111101110001111002000001001020000131001010000000

Choristodera

211000010101010111(01)00?1100000010?(01)2111110110?(01)001001011101110111?0011?0??110010001000011010010?0??10110000010100001110100010(01)100110010001100?00111000101?00010001(02)1120010010(01)0?101110?10111010101001001000100010011010000000

*Macrocnemus*

11000011?1000?01?11?0?1?01?000001021111101210?????0??0?1?1011?11?01?1110?1?0??0??00???0101001?1001101?0000010110001012110??00100101?10011?00000011000110000000001?122001?00011110111000011101000000100102000?131?01010000000

*Hovasaurus*

????????????????????0??00?101?0??1?0?0??002011??00?10????01???01?0???1????????????????2?????1???????????????0?0???00000?0??0???0?01?10002??0?000010?????10001000120?2?010?10000?01?10000?0??101020???00000000?300?100?001000

'*Anaro-Dactylosaurus*'

1000001010000??2010?0??00100101111011111022010111?0?10?1011101??1???11?100111(01)?0111???1?0100100111?11000(01)001021000101011101010001(12)1020002?(01)000011101211?0200(01)1101(01)0(12)(12)001201000110110000110000011(01)10111(01)101111130112(01)10000000

*Simosaurus*

2000001010100??2010?0??00000001011121111022010111?010011001101??1?1?10?1000111?011000211011010?11??110001101121100111011101010?111102000??00?001100121000200111011122001201?0011?110000110000211?10111?10111012011212000?000

*Placodus*

0010011001010??2010?11?00101100111101111002011??1??10010010101??1?1?110110200110110?01001101111000111101101102101000101101011001101020002?100111110111000000101010122001201000110111000110000(02)11220111010110112010?110000000

Pistosauridae

10?0001010100??2011?0??01100001011131011012011??1?0?0111001101??1???11?1001011?0110?0?1?0???10?11??1000011011210001110??1010100?1?102(01)0???00?0(01)11101?1??0200?11011112011001010(01)?01?000?????001??0(012)1111??01111?2?1?21?0000000

*Askeptosaurus*

100001110010000100100?001010101010001111012101??0101?11101111?11?01?1?0001?0?010000112110001100111?01000000112100010101101101000101010?000000?00100001000000000010121011001000?101110000111000102?11100000000030112110000000

*Clarazia*

0011001100100???001?0????11011100?00?1110122?1??010??111?1111???????1?000110?010??0?1?0111011?000??311000011101110?0?0110?0010?0?01010?01?000???110?0100000000?0????2001?00?00??11110?01110000110?011?0?0?000?00?1??10000010

*Thalattosaurus*

1011001100100???011?01?0?110?1100?00?111012201??010??111?1111???????1???01?0?01???0?1?111101100011121100001?12011?1010?00?10???00??01????????????????????100?00????????1?????????01??????????01???111????0?00?3011??1000??00

*Helveticosaurus*

00?1001?1?????0?000?0?1?0????1??11???0????????????????????????????????????????????????0?0?001??000?00??0000?????00?0?01101?101001?10200?1?00?10011000100020010001?111001?010000100110??111000?1132010????011103011202?000000

*Largocephalosaurus*

00?0001?00000??201110?10111000001111011102?011??010??0???0010??????1?100?1?01?1?0?0???110100???00??01?0(01)0?0??21?01101?1110?101000(01)??2(23)20001010111101210002001?101(02)1210110010(01)00?0011??0(01)100??(02)10100111010111113011?11000?000

*Sinosaurosphargis*

00?0001000000???01110?1??1110?00??100111022011???????0????010??????1??00??1010100?0002110000???0???01?00010?121001?00?1??2?1????????202???10102111012???????1010????10110000??0??????????????2???0011????????10??1?1?000?000

*Wumengosaurus*

10?00?0100000???01110??011?01010?100?0110221?0111??????1?01?0???????1????0?11??01?1???0?01001?0000??1?(01)0000?????01101?100??01000110020000?(01)0000?10?1210002001?(01)01(03)0220010010100?0(01)100201100000112111110101111130112110000000

*Nanchangosaurus*

10?0?01?0?000?0100110?00011110011110101102210011001?00???00?0???????????????1?????????0??100???0??????02????????0?1?1??10?00100000002010111012121100?1??0000?00113122?11?1?00???001??????????01020?1110??11?1?3011?12010002?

*Hupehsuchus*

10?0?01101000?0100111?00011110011110001102210011001??0???00?0????????????0??11?0??1???0?0100???0?0????02????12100?101?110?00100000002010111012121100?1??0000?001131221111110001?001001001000001020?111010111113011212010{01}02?

*Chaohusaurus*

10?0011101001?0100111?00112000010100101102210011001??0???0110?????????0??0?01?100?1???0?00001??001?01?100001121010100?110?0210100(01)101000010000021000210?0000000113122111(01)011000?101000011100001(01)001111010111113011(012)120010000

*Utatsusaurus*

10?0011101001?0101110?0?112100011110111102010011001??0???001?????????????0?01010??1???1100001?0011?01?100001111000101?110??2101?010010000?000002110001000000000113122?11(01)011000?10100100110000100011110101011110112120010000

*Cartorhynchus*

00?0011101001?0100010?00002000100100111102210011001??0?1?01?0?????????????????????????0?00001??000?01?1210?1121010100?110?0210??0?10100001100302110001000000000113122111?0?10?0?001??????????01?0101110???111?3011?12101010?

*Sclerocormus parviceps*

00?0011??1001?0100?11?100001001001000111{01}2?0??0?00????????1???????????????????????????1???0???????????12????????????0??1???2100?0??0100?0?10131211???1???0???0?1?????????0?100??001??????????01?00011?0?0?11?11??1??211111??

*Odontochelys*

20?000?010000???001?1?1???10010?1?2001110???????00???????????????0?????0???0?00001????0?00??????????1?0?0?1111010??0????0???????00??101???0???0?1????????100?0000???2001??1000??0111???0111??010?0000?1111???000?0?000???000

*Sclerocormus* cf. *S. parviceps*

01?0011101001?010011{01}?1000010010010001110220000100???????01?0????????????0??1?????????1000001??000?01?1210?112101010??1????2????0?1?100???10131{02}110011000010100113122111(01)0?1?????????????????0???00111???1????1????12110?111

*Eusaurosphargis*

?0?0?0??????0???0??????0??????0???10?0?????????????????????????????????????0?00?????????0??????????01??01???021?011?1010?1?1??0?10???{12}{01}0??00001111010100020000001?02?0?00010101?0111000110001?1112000?1101011130??212000?000

*Omphalosaurus*

?????1???1001??100{01}??????0?10???????????{01}?????????????????1?????????????????1??02?????100??01???00?01???1???121010?0???????2????0?1?100???10???{02}???????????????????????????????????????????????????11????????????????00???11
