# Supplementary material for: A globally distributed durophagous marine reptile clade supports the rapid recovery of pelagic ecosystems after the Permo-Triassic mass extinction
Source: Commun Biol. 2022 Nov 14;5:1242. doi: 10.1038/s42003-022-04162-6 (PMC9663502; doi:10.1038/s42003-022-04162-6)
Supplement: Supplementary file 7 — Reporting Summary [file 42003_2022_4162_MOESM7_ESM.pdf]

nature portfolio

Corresponding author(s): Jun Liu

Last updated by author(s): Jun 13, 2022

Reporting Summary

Nature Portfolio wishes to improve the reproducibility of the work that we publish. This form provides structure for consistency and transparency in reporting. For further information on Nature Portfolio policies, see our Editorial Policies and the Editorial Policy Checklist.

Statistics

For all statistical analyses, confirm that the following items are present in the figure legend, table legend, main text, or Methods section.

n/a	Confirmed	
☒	☐	The exact sample size (*n*) for each experimental group/condition, given as a discrete number and unit of measurement
☒	☐	A statement on whether measurements were taken from distinct samples or whether the same sample was measured repeatedly
☒	☐	The statistical test(s) used AND whether they are one- or two-sided<br>*Only common tests should be described solely by name; describe more complex techniques in the Methods section.*
☒	☐	A description of all covariates tested
☒	☐	A description of any assumptions or corrections, such as tests of normality and adjustment for multiple comparisons
☒	☐	A full description of the statistical parameters including central tendency (e.g. means) or other basic estimates (e.g. regression coefficient) AND variation (e.g. standard deviation) or associated estimates of uncertainty (e.g. confidence intervals)
☒	☐	For null hypothesis testing, the test statistic (e.g. *F*, *t*, *r*) with confidence intervals, effect sizes, degrees of freedom and *P* value noted<br>*Give P values as exact values whenever suitable.*
☒	☐	For Bayesian analysis, information on the choice of priors and Markov chain Monte Carlo settings
☒	☐	For hierarchical and complex designs, identification of the appropriate level for tests and full reporting of outcomes
☒	☐	Estimates of effect sizes (e.g. Cohen's *d*, Pearson's *r*), indicating how they were calculated

Our web collection on statistics for biologists contains articles on many of the points above.

Software and code

Policy information about availability of computer code

Data collection	Avizo 2019.4
Data analysis	TNT ver 1.5 R; package StatMatch

For manuscripts utilizing custom algorithms or software that are central to the research but not yet described in published literature, software must be made available to editors and reviewers. We strongly encourage code deposition in a community repository (e.g. GitHub). See the Nature Portfolio guidelines for submitting code & software for further information.

Data

Policy information about availability of data

All manuscripts must include a data availability statement. This statement should provide the following information, where applicable:

- Accession codes, unique identifiers, or web links for publicly available datasets
- A description of any restrictions on data availability
- For clinical datasets or third party data, please ensure that the statement adheres to our policy

We declare that the datasets supporting this article have been uploaded as part of the supplementary material.

Human research participants

Policy information about studies involving human research participants and Sex and Gender in Research.

Reporting on sex and gender	NA
Population characteristics	NA
Recruitment	NA
Ethics oversight	NA

Note that full information on the approval of the study protocol must also be provided in the manuscript.

Field-specific reporting

Please select the one below that is the best fit for your research. If you are not sure, read the appropriate sections before making your selection.

☐ Life sciences ☐ Behavioural & social sciences ☒ Ecological, evolutionary & environmental sciences

For a reference copy of the document with all sections, see nature.com/documents/nr-reporting-summary-flat.pdf

Ecological, evolutionary & environmental sciences study design

All studies must disclose on these points even when the disclosure is negative.

Study description	We discovered a new fossil specimen of Sclerocormus, an extinct ichthyosauriform, and revised the ichthyosauriform systematics based on new morphological observation.
Research sample	Sclerocormus cf. parviceps (HFUT MJS-16-012), collected from Majiashan quarry in Chaohu, Hefei, Anhui Province, China. Ontogenetic stage and sex of the specimen are unknown.
Sampling strategy	The new specimen studied was collected from Majiashan quarry in Chaohu, Hefei, Anhui Province, China.
Data collection	NA
Timing and spatial scale	NA
Data exclusions	NA
Reproducibility	NA
Randomization	NA
Blinding	NA

Did the study involve field work? ☒ Yes ☐ No

Field work, collection and transport

Field conditions	These data were not collected since they are irrelevant with the study performed here.
Location	Majiashan quarry in Chaohu, Hefei, Anhui Province, China
Access & import/export	The permit is not needed when the specimen was collected by hand without involving the machine, following the Chinese regulations of fossil protection.
Disturbance	No disturbance caused

Reporting for specific materials, systems and methods

We require information from authors about some types of materials, experimental systems and methods used in many studies. Here, indicate whether each material, system or method listed is relevant to your study. If you are not sure if a list item applies to your research, read the appropriate section before selecting a response.

Materials & experimental systems

n/a	Involved in the study
☒	☐ Antibodies
☒	☐ Eukaryotic cell lines
☐	☒ Palaeontology and archaeology
☒	☐ Animals and other organisms
☒	☐ Clinical data
☒	☐ Dual use research of concern

Methods

n/a	Involved in the study
☒	☐ ChIP-seq
☒	☐ Flow cytometry
☒	☐ MRI-based neuroimaging

Palaeontology and Archaeology

Specimen provenance	The specimen studied was collected from Majiashan quarry in Chaohu, Hefei, Anhui Province, China. The permit is not needed when the specimen was collected by hand without involving the machine, following the Chinese regulations of fossil protection.
Specimen deposition	The specimen is deposited in the Geological Museum of Hefei University of Technology, Hefei, China and permit a free access to other researchers.
Dating methods	NA

☒ Tick this box to confirm that the raw and calibrated dates are available in the paper or in Supplementary Information.

Ethics oversight	No ethical approval was required since there is no such requirement for paleontological research like this following the Chinese regulations of fossil protection.

Note that full information on the approval of the study protocol must also be provided in the manuscript.
